# Fengycin A Analogues with Enhanced Chemical Stability and Antifungal Properties

**DOI:** 10.1021/acs.orglett.1c01387

**Published:** 2021-06-02

**Authors:** Diana Gimenez, Aoife Phelan, Cormac D. Murphy, Steven L. Cobb

**Affiliations:** † Department of Chemistry, Durham University, South Road, Durham DH1 3LE, United Kingdom; ‡ School of Biomolecular and Biomedical Science, University College Dublin, Belfield, Dublin 4, Ireland

## Abstract

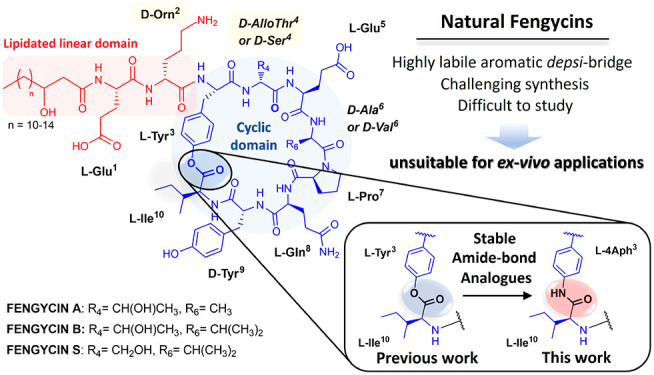

Fengycins are cyclic lipo-depsipeptides produced by *Bacillus spp.* that display potent antifungal properties but are chemically unstable. This instability has meant that no total synthesis of any fengycin has been published. Here we report the synthesis of fengycin A analogues that display enhanced antifungal properties and chemical stability under both basic and acidic conditions. The analogues prepared also demonstrate that the fengycin core structure can be modified and simplified without the loss of antifungal activity.

Plant pathogens, and in particular fungal diseases, pose an increasing risk to global food security.^1−3^ An important part of the crop protection arsenal is *Bacillus subtilis* strains, which are used as biological control agents (BCAs) (Serenade).^4−6^ Bioactive *Bacillus spp*. are able to secrete, as an innate response to external microbiota stimuli, potent antimicrobial metabolites that include the cyclic lipopeptides (CliPs) from the iturin, surfactin, and fengycin families (Figure 1).^5,7−13^ Fengycins are the dominant CliPs in *Bacillus spp*.^5,14,15^ and are active against a range of phytopathogenic fungi,^12,16−19^ causing cell lysis and leakage through binding with the plasma membrane.

**Figure 1:**
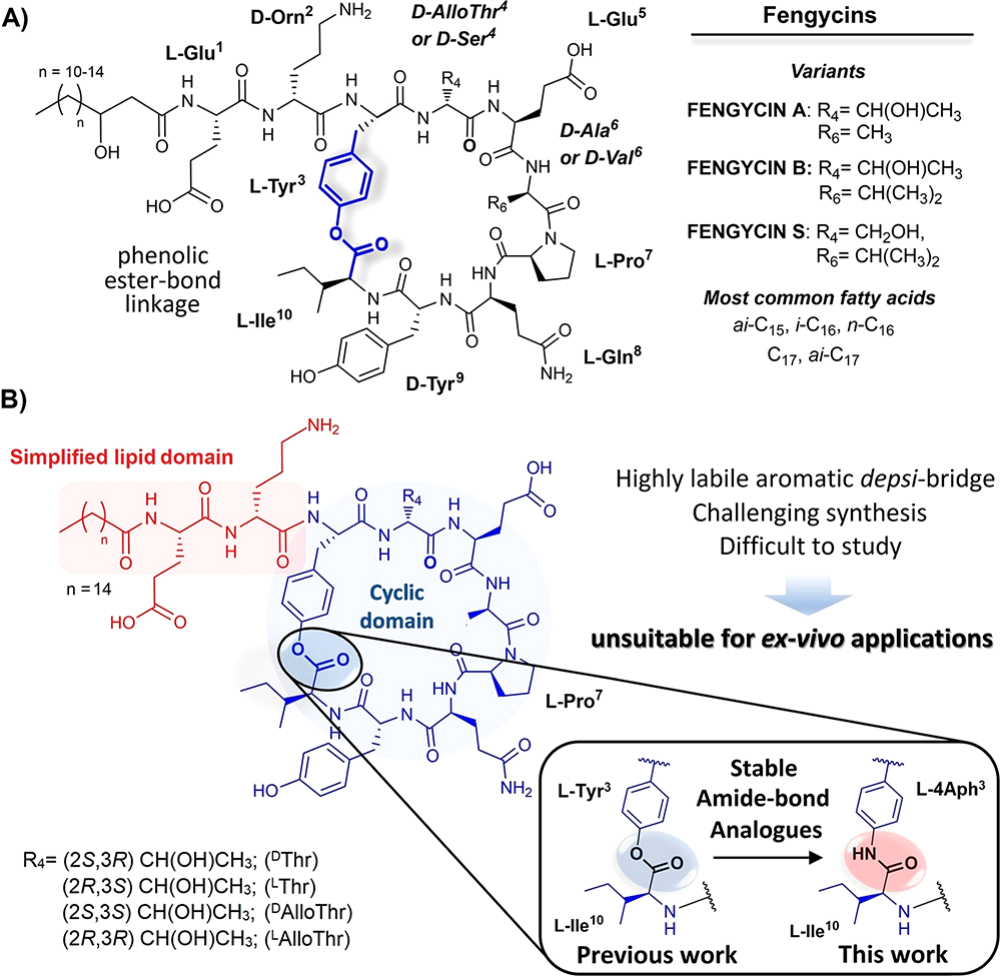
(A) General structure of fengycin. (B) Proposed approach for fengycin stabilization.

While most research on BCAs has focused on the direct application of live bacteria, the effects of a range of environmental factors (i.e., soil type and humidity) can produce broad inconsistencies in their performance within the field.^20^ A further disadvantage relates to the fact that antifungal BCAs act slowly when compared to typical pests, and therefore only give a very time-limited protection to crops.^21^ In light of these factors the application of individual bioactive CliPs, rather than the entire living organisms, would be an attractive option for the development of new crop protection agents. However, the problem with this strategy is that isolation and/or chemical synthesis of certain CliPs, like fengycin, has proven to be challenging.

Contrary to iturins and surfactins, fengycins display a hydrolytically susceptible aromatic *depsi* bond between Tyr^3^ and Ile^10^_,_ which significantly compromises the structural integrity of its cyclic core (Figure 1A).^22,23^ This intrinsic lack of chemical stability means that fengycins are technically challenging to prepare via chemical synthesis.^24,25^ This is clearly highlighted by the fact that to date no synthetic strategy has succeeded in delivering a completely natural fengycin peptide and, only very recently, a solid phase peptide synthesis (SPPS) approach which enabled the synthesis of several fengycin analogues was reported.^26^ While this marked a significant advance for the field, the reported approach only afforded modest product yields and it did not address the issue of the fengycin peptides’ innate instability.

To address the challenges associated with both the synthesis and stability of fengycins, we proposed to prepare a series of modified and simplified analogues (Figure 1B). To solve the issue of its chemical stability we hypothesized that fengycin derivatization into a lactam-bridged cyclopeptide, rather than through its natural ester functionality, would enable access to more stable cyclic analogues. This approach has been shown previously in the literature to help improve the chemical stability of a range of cyclic peptides.^27−29^ Second, in order to simplify and reduce the associated cost of the synthesis we sought to replace the d-allo-Thr residue at position R_4_ and also remove the chiral hydroxyl center from the lipid tail. Herein, we report an efficient SPPS route that allows ready access to fengycin A analogues with enhanced antifungal and chemical stability when compared to the natural product.

Not constrained by the limitations imposed by the natural *depsi*-bridge, we sought to design possible SPPS routes that could achieve efficient cyclization yields and enable flexible peptide diversification *on resin* (Figures 2 and 3). We hypothesized that a strategy based on an early stage peptide cyclization, rather than a late-stage peptide macrolactamization, would be beneficial due to the lower conformational flexibility of the peptide chain, allowing for increased linear to cyclic product conversions.

**Figure 2:**
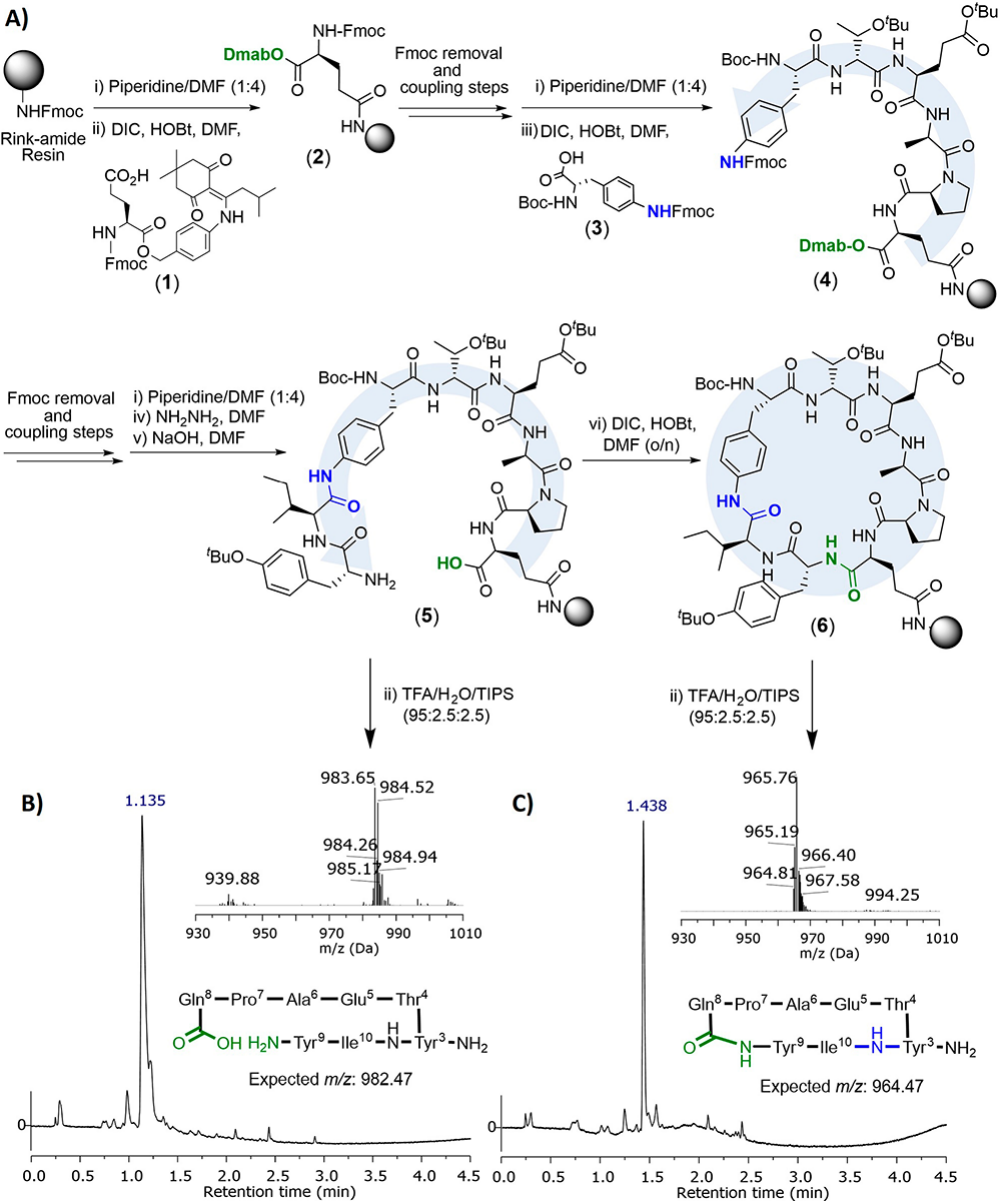
(A) Synthesis of cyclic peptidyl-resin **6**. LC/(ESI^+^)MS traces of cleaved sample aliquots of **5** before (B) and after cyclization (C) showing quantitative conversion to the cyclic product **6** (λ= 254 nm).

**Figure 3:**
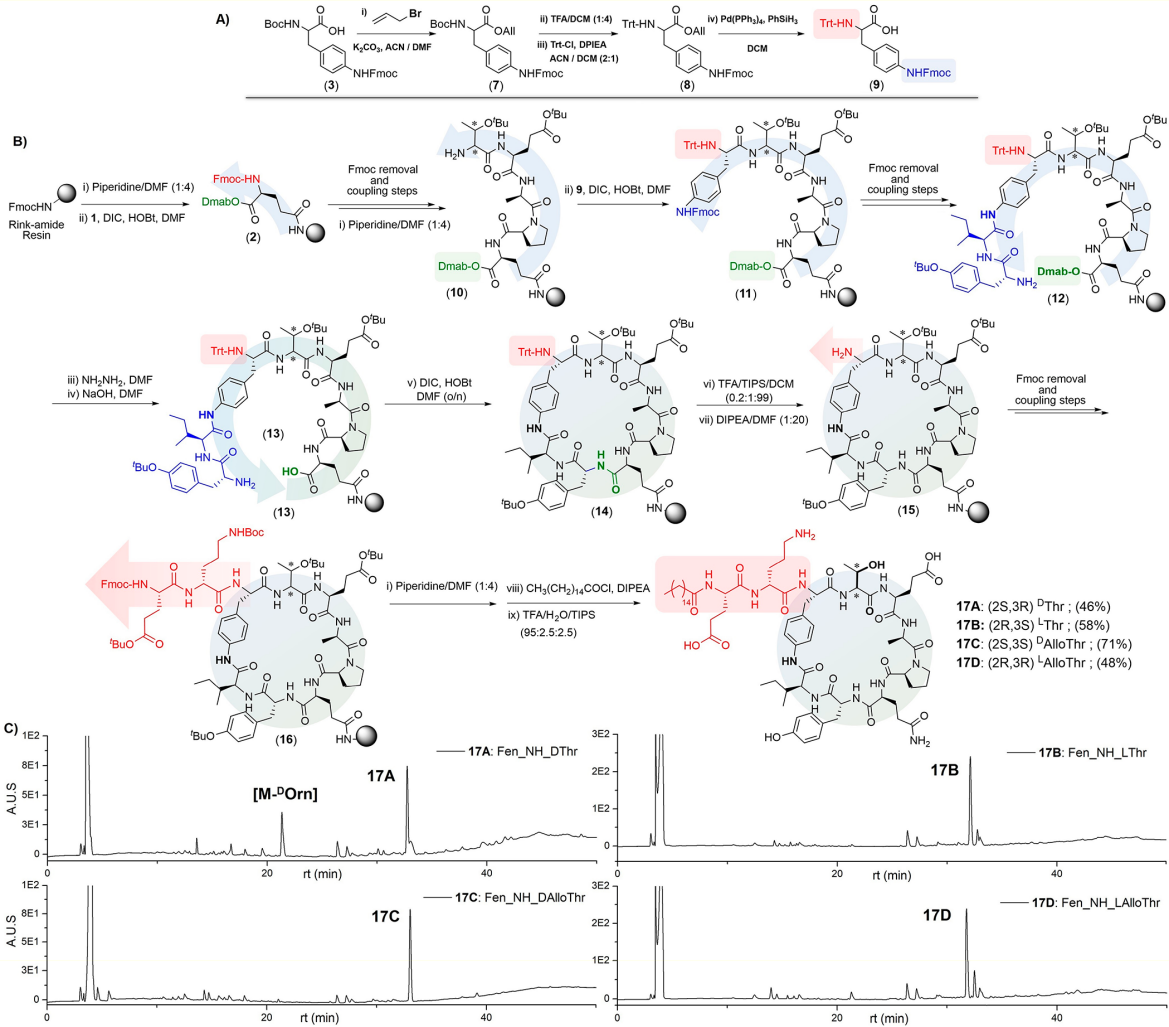
(A) Chemical synthesis of Trt-l-4(NFmoc)Aph-OH (**9**). (B) Complete total SPPS strategy, based on a quaternary Fmoc/Trt/Dmab/(*t*Bu/Boc) protection scheme, developed in this study for the preparation of lactam fengycin analogues **17A**–**D**. Product purities of the crude materials are given as analyzed by RP-HPLC (λ= 220 nm). (C) HPLC traces of crude peptides **17A**–**D** (λ= 220 nm).

On this basis, we first designed a synthetic route for the cyclic core, using a Fmoc/(*t*Bu/Boc)/Dmab protection scheme, that could take advantage of the presence of a natural l-Gln residue in the peptide structure to install a side-chain anchor to the resin (Figure 2). Under this strategy, Fmoc-Glu(OH)-ODmab (**1**) is thus attached in the first step of the synthesis to the Rink-amide resin. Dmab protection was selected as it is orthogonal to base-labile Fmoc and acid-labile *t*Bu/Boc protecting groups and its selective removal can proceed quantitatively in the presence of hydrazine (2–5% *v*/*v* in DMF).^30^ Other options, such as allyl esters, have proven on occasion in our hands to be difficult to deprotect.^31,32^

To test the suitability of this approach, we synthesized model peptidyl-resin **5** following standard Fmoc/*t*Bu chemistry procedures. Peptide **5** mimics the sequence of the fengycin cyclic domain but for the ^3^Tyr-^10^Ile *depsi*-bridge, which was replaced for an amide bond linkage by using Boc-4-(Fmoc-amino)-l-phenylalanine (**3**) (Figure 2A, see Supporting Information for complete experimental details). Then, we proceeded to evaluate the efficiency of the intramolecular cyclization step (Figure 2B,C). For this, **5** was incubated overnight in the presence of DIC/HOBt (3 mol equiv each; rt) and sample aliquots of the resins before (**5**) and after cyclization (**6**) cleaved in the presence of TFA/H_2_O/TIPS, 95:2.5:2.5% *v*/*v*. Satisfyingly, analysis of the crude materials by LC/(ESI^+^)MS spectrometry at λ = 254 nm, characteristic of the aromatic residues present, confirmed quantitative conversion of the linear peptide **5** (*m*/*z* = 983.6 Da, *t*_R_ = 1.1 min; [M + H]^+^; Figure 2B) to the expected cyclic product **6** (*m*/*z* = 965.7 Da, *t*_R_ = 1.4 min; [M + H]^+^; Figure 2C).

Next, we turned our attention to addressing the complexity of the branched fengycin structure (Figure 3). For this, peptidyl-resin **2** was resynthesized and the peptide sequence extended until the key residue where the peptide bifurcates (**10**, Figure 3B). At this critical point, we had anticipated the need for a protected 4Aph derivative that could enable both the temporal protection of the peptidyl-resin N-terminus and the controlled propagation and ring closure of the peptide through its aniline functionality.

We also considered that such a derivative must allow for subsequent peptide Ct → Nt elongation by means of conventional Fmoc amino acids, to prevent posterior racemization and to minimize the need for custom-made materials. Chemical orthogonality to the Rink-amide C-terminus, side chain Boc/*t*Bu, and Dmab protecting groups was also needed in the protecting group approach selected. Given the aforementioned factors we opted to synthesize the NTrityl/NFmoc protected amino acid Trt-l-4(NFmoc)Aph-OH (**9**, Figure 3A).

As described, in Figure 3A, amino acid **9** could be synthesized in 4 steps from its commercially available Boc-4-(Fmoc-amino)-l-phenylalanine precursor (**3**). First, the carboxylic functionality in **3** was converted to its allyl ester using K_2_CO_3_ (1 mol equiv) and allyl bromide (1.5 mol equiv).^33^ Next, the Boc group was removed in TFA (20% *v*/*v* in DCM) and the Trt protection of the N-terminus achieved by slow addition of DIPEA (4 mol equiv) and Trt-Cl (1.5 mol equiv) in CH_3_CN/DCM 2:1. Lastly, selective removal of the allyl protecting group in **8** with Pd(PPh_3_)_3_ and PhSiH_3_ in DCM afforded the expected Trt-l-4(NFmoc)Aph-OH (**9**) with sufficient purity that it could be readily incorporated into the peptide synthesis without any further purification (see Supporting Information for further details). Once **9** was coupled to the growing sequence, **11** was further elongated using standard procedures to yield target peptide **13**, which was then cyclized in situ (Figure 3B).

With the fengycin cyclic core in hand, we then proceeded to complete the lipidated *N*-terminus. For this, the Trityl group in **14** was removed using a diluted solution of TFA/TIPS in DCM (0.2/1% *v*/*v*, 1 min, 5×) and the resulting peptide neutralized in DIPEA/DMF (5% *v*/*v*, 60 min). d-Orn (2.5 equiv) and l-Glu (5 equiv) were then sequentially incorporated via DIC/HOBt assisted couplings to complete the synthesis of the peptide component of the target. Finally, incorporation of the lipid tail was achieved by acylation of Fmoc deprotected resin (**16**) with palmitoyl chloride in the presence of DIPEA.

Gratifyingly, when the corresponding peptidyl-resin was cleaved we obtained the fengycin analogue **17A** (crude yield of ∼50%; Figure 3C). It is worth noting that, as seen in the cyclization of test peptide **5**, no significant traces of the linear peptide or dimeric species could be found upon analysis of the crude material.^22^ The only byproduct observed was a d-Orn depleted analogue produced *after* cyclization due to the modest excess of this amino acid employed during the synthesis (21%, [M-114] Da, Figure 3; see also supporting Figures S19–20).

Similar or improved results were obtained when this methodology was employed to prepare **17B**–**17D**, where we replaced the initial d-Thr residue at position 4 by the fengycin A naturally occurring d-allo-Thr and also their corresponding enantiomers (Figure 3C). Previous reports have shown that the chirality of the residues within the cyclic core of natural fengycin affects the ease of ring formation.^22,26^ However, our results in the synthesis of analogues **17B** and **17D**, using l-amino acids, show that this is not the case for fengycin lactam derivatives, as all of them could be synthesized with similar overall efficacy (50–70% crude purities, Figure 3C and Figures S23–29). Overall, the synthesis of **17A**–**D** clearly demonstrates the suitability of our synthetic approach for the convenient SPPS of fengycin cyclic lipopeptide amide analogues.

With peptides **17A**–**D** prepared, we moved to investigate the differences in their chemical stability in comparison to that of the natural product. To this end, biosynthetic fengycin expressed from *Bacillus spp.* was purified by RP-HPLC and the major product, C_16_-fengycin B, was isolated and characterized (see Figures S32–S36). Hydrolysis studies were carried out with this compound due to the challenges in isolating enough pure fengycin A. We then selected hydrolysis conditions for our stability studies (50 mM NaOH for basic hydrolysis and 50% *v*/*v* TFA/H_2_O for acidic degradation; see Supporting Information for details).

When natural fengycin (C_16_-fengycin B) was incubated at room temperature in the presence of 50 mM NaOH, complete peptide degradation was observed within 5 min (Figure S37). In comparison, all of the amide-based fengycins analogues were found to be remarkably stable under the same conditions even for periods as long as 18–24 h (76–90% intact peptide, see Figures S39, S41, S43, S45). Acidolytic hydrolysis in the presence of TFA also revealed significantly different degradation profiles for the natural product (complete degradation in 12 h; Figure S38) when compared to the modified amide-*bridged* analogues (75–90% intact peptide, Figures S40, S42, S44, S46). The results obtained highlight the superior chemical stability exhibited by all of the amide analogues of fengycin. They also demonstrate that modification of the labile natural *depsi* Tyr^3^-O-Ile^10^ ester bond is a useful route by which to enhance the half-life of this family of bioactive molecules.

While the enhanced chemical stability of the fengycin analogues was welcomed, it was expected that the change to a lactam bridge would impact the biological activity; thus, bioassays were conducted to examine the effect of **17A**–**D** on the growth of the fungus *Fusarium graminearum*. This fungus was selected as it is known to be sensitive to the natural lipopeptide.^34^Figure 4 shows the results from growth measurements in well-plates in the absence and presence of the natural and synthetic fengycins.

**Figure 4:**
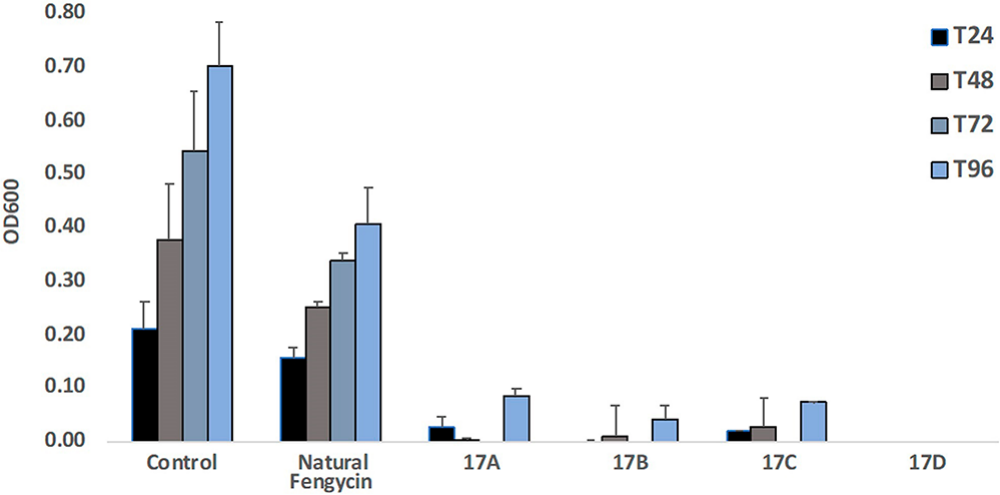
Effect of natural and synthetic fengycins on the growth of *Fusarium graminearum*. Fungal growth was measured in the absence (control) or presence of 500 μg/mL fengycin. *t* tests confirmed that all differences between natural and synthetic fengycins were significant (*p* < 0.05).

The results presented in Figure 4 show that the synthetic fengycins (**17A**–**D**) inhibited growth to a much greater degree than natural fengycin isolated from *Bacillus* CS93. In fact, **17D** was found to completely inhibit fungal growth.

In conclusion, we have developed an efficient synthetic route for the preparation of lactam-containing fengycin analogues. Given its modular approach and compatibility with readily available Fmoc amino acids, this method can be easily adapted to give access to a range of new fengycin derivatives. The fengycin analogues prepared in this study displayed enhanced antifungal properties over the naturally occurring material. Importantly, in addition to the enhanced antifungal properties, replacement of the natural depsi-bridge by an amide-bond linkage was found to significantly enhance their chemical stability under both basic and acidic conditions. Finally, this work demonstrates that the fengycin core structure could be modified (e.g., via amino acid substitution) and the lipid tail structure simplified without the loss of antifungal activity. This discovery combined with the SPPS approach reported herein will offer new opportunities to further develop this class of molecules as anti-infective agents for applications in both medicine and agriculture.

## Supporting Information Available

Experimental procedures, product characterization, HPLC stability studies data, and microbiological experimental description (PDF)
