# Supplementary material for: Fengycin A Analogues with Enhanced Chemical Stability and Antifungal Properties
Source: Org Lett. 2021 Jun 2;23(12):4672–6. doi: 10.1021/acs.orglett.1c01387 (PMC8289291; doi:10.1021/acs.orglett.1c01387)
Supplement: Supplementary file 1 — ol1c01387_si_001.pdf [file ol1c01387_si_001.pdf]

Fengycin A analogues with enhanced chemical stability and antifungal properties

Diana Gimenez,[†] Aoife Phelan,[‡] Cormac D. Murphy[‡] and Steven L. Cobb *,[†]

[†]Durham University, Department of Chemistry, South Road, Durham, DH1 3LE, United Kingdom.

[‡] School of Biomolecular and Biomedical Science, University College Dublin, Belfield, Dublin 4, Ireland.

[†]Corresponding Author E-Mail: s.l.cobb@durham.ac.uk

Table of Contents

Materials and General Methods **2**

Synthesis of NTrt(NFmoc)Aph-OH (9) **3**

NBoc(NFmoc)Aph-OAll (7) *3*

NBoc(NFmoc)Aph-OAll (8) *5*

NTrt(NFmoc)Aph-OH (9) *12*

Solid-Phase Peptide Synthesis **19**

Solid-Phase Synthesis of Cyclic peptide 6 .... *19*

Solid-Phase Synthesis of Cyclic NH Lipopeptides 17A-17D *20*

Chemical Stability Studies **31**

Natural Fengycin isolation and characterization *31*

General procedure for sample hydrolysis and sample analysis *36*

HPLC data *37*

Microbiological experiments **47**

Additional references **47**

Materials and General Methods

All peptide synthesis was performed manually in a polypropylene syringe fitted with a polyethylene porous disc. Rink-amide resin and amino acid derivatives were purchased from NovaBiochem. Other chemicals, including coupling reagents, were purchased from Fluorochem, Alfa Aesar or Sigma-Aldrich and used without further purification. All solvents used were purchased from Fisher Scientific Ltd. and were synthesis grade except for CH_3CN, which was HPLC grade.

Preparative thin layer chromatography was undertaken using Analtech Uniplate silica gel chromatography plates containing a fluorescent indicator (254 nm) (20x20 cm, 1500 µm). Flash column chromatography was performed using silica gel (60 Å pore size, 40 – 63 µm particle size, 230 – 400 mesh).

Liquid chromatography (LC)-mass spectrometry (ESI-MS) analyses were performed on a Acquity UPLC BEH C_{18} 1.7µm (2.1mm x 50mm) column using a Waters Acquity UPLC system equipped with a photodiode array detector, providing absorbance data from 210 nm to 400 nm. A gradient with eluent I (0.1% HCOOH in water) and eluent II (0.1% HCOOH in acetonitrile) rising linearly from 5 to 95% of II during *t*= 0.2–4.0 min was applied at a flow rate of 0.6 ml min^{-1} after 0.2 min of 95% solvent I equilibration.

Analytical HPLC analysis were performed on a X-Bridge C_{18} column (5.3µm, 4.6 x 100 mm, 40 °C) using a Pelking-Elmer 200 series lc system supplied with auto-sampler, UV/Vis detector and a Peltier column oven. A linear gradient rising from eluent III (95:5:0.05% *v/v* H_2O:MeCN:TFA) to 100% of eluent IV (5:95:0.03% *v/v* H_2O:MeCN:TFA) over 40 min was applied at a flow rate of 1 ml min^{-1}. Detection was performed in all cases at λ = 220 nm, unless otherwise stated. Sample purity has been estimated in the basis of the integrated area under the corresponding chromatographic peaks.

Peptide purification was performed by means of semi-preparative HPLC using a Discovery Bio wide pore C_{18}-5 column from Supelco (5 µm, 25cm × 10 mm) in a Pelking-Elmer 200 lc pump coupled to a Waters 486 tunable absorbance detector settled up at λ = 220 nm. A gradient using eluent V (95:5:0.1% H_2O:MeCN:TFA) and eluent VI (5:95:0.1% H_2O:MeCN:TFA) was applied where solvent VI was increased linearly from 30 to 100% over 40 min at a flow rate of 2.3 ml min^{-1}.

High-resolution QToF-LC/MS (HRMS) analyses were performed on a Acquity UPLC BEH C_{18} 1.7µm (2.1mm x 50mm) column using a Waters Acquity UPLC system coupled to Micromass QToF Premier mass spectrometer, also equipped with a photodiode array detector providing absorbance data from 210 nm to 400 nm. A gradient with eluent I (0.1% HCOOH in water) and eluent II (0.1% HCOOH in acetonitrile) rising linearly from 0 to 99% of II during *t*=0.0–5.0 min was applied at a flow rate of 0.6 ml min^{-1}.

Tandem MS/MS analyses were performed in a Synapt G2s HDMS mass spectrometer equipped with an Acquity UPLC system as described above. A gradient with eluent I (0.1% HCOOH in water) and eluent II (0.1% HCOOH in acetonitrile) rising linearly from 5 to 95% of II during *t*=0.0–5.0 min was applied at a flow rate of 0.4 ml min^{-1}.

NMR spectra was recorded on a Varian VNMRS-700 NMR spectrometer at 298 K. ^{1}H-NMR has been obtained at 400 or 700 MHz using 4-8 scans with a relaxation delay of 10 s between them. ^{13}C-NMR has been obtained at 176MHz (1000-2156 repetitions, 0.7-3 seconds of relax. delay) and bi-dimensional 1H-1H-COSY, ^{1}H-^{13}C-HCSQCAD and ^{1}H-^{13}C-HCSQCAD experiments have been run with minimum spectral width of 6,000 Hz in both dimensions acquiring 2 transients with 2 x 512 increments. Final minimum FT size = 2048 x 2048 points. All data has been processed using Mestrenova® software, and chemical shifts are reported in p.p.m., relative to residual solvent peaks as internal standards (δH, $CDCl_3$: 7.26 p.p.m.; δC, $CDCl_3$ 77.16 p.p.m., δH, CD_3CN 1.94 p.p.m.; δC, CD_3CN 1.32 p.p.m.; δH, CD_3OD: 3.34 p.p.m.; δC, CD_3OD 49.00 p.p.m.). The following multiplicity abbreviations are used: (*d*) doublet, (*dd*) double doublet, (*td*) triple doublet, (*m*) multiplet, and (*br*) broad peak.

Synthesis of NTrt(NFmoc)Aph-OH (9)

Trt(NFmoc)Aph-OH was synthetized according to Figure 3A in 4 steps from its commercially available Boc-(NFmoc)Aph-OH derivative (Fluorochem). The procedure is detailed next.

N-Boc-(NFmoc)Aph-OAll (7):

To N-*tert*-butoxycarbonyl-N-(9-fluorenylmethoxycarbonyl)-L-4-aminophenylalanine, **3**, (5.0 g, 9.9 mmol) and K_2CO_3 (1.38 g, 9.9 mmol) in MeCN:DMF 9.5:0.5 (10 mL) was added allyl bromide (1.2 mL, 14.8 mmol) at 0 ºC. The reaction mixture was stirred at room temperature for 2 h, until no further evolution was observed by TLC chromatography. Then the solvent was removed under *vacuum* and the crude mixture was dissolved in EtOAc (100 mL) and washed with water (2 x 50 mL), brine and dried over $MgSO_4$. Evaporation of the solvent, afforded 4.9 g of crude N-*tert*-butoxycarbonyl-N-(9-fluorenylmethoxycarbonyl)-L-4-aminophenylalanine allyl ester, **7**, which was taken forward without purification. ESI(-)-LC/MS: 1129.34 $[2M+CO_2H-]^-$; 587.30 $[M+CO_2H-]^-$; 319.30 $[M\text{-}Fmoc\text{-}H]^-$. HRMS (ESI) m/z: [M - H]- Calcd for C32H33N2O6 541.2339; Found: 541.2328

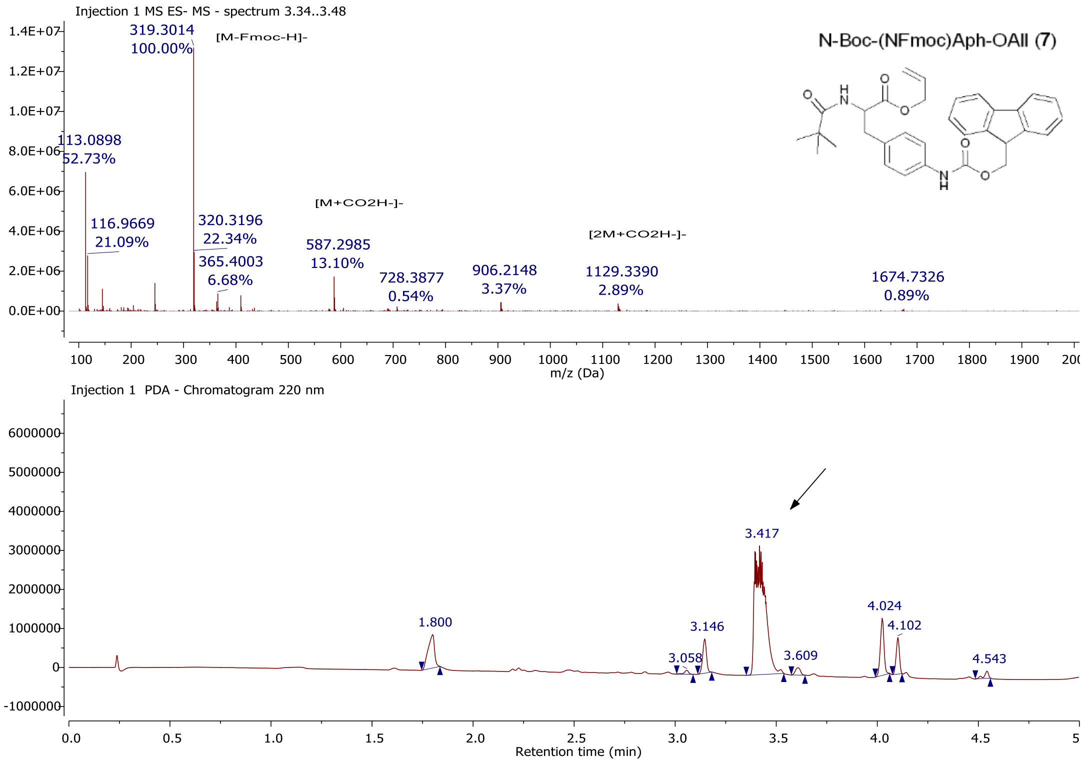

Figure S1: (ESI-)LC/MS trace at λ = 220 nm of crude compound **7**.

Elemental Composition Report

N-Boc-(NFmoc)Aph-OAll (**7**)

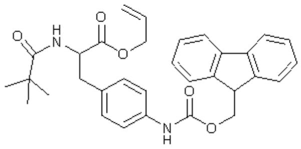

Single Mass Analysis

Tolerance = 5.0 PPM / DBE: min = -1.5, max = 100.0
Element prediction: Off
Number of isotope peaks used for i-FIT = 5

Monoisotopic Mass, Even Electron Ions
4328 formula(e) evaluated with 25 results within limits (up to 500 closest results for each mass)
Elements Used:
C: 0-50 H: 0-50 N: 0-7 O: 0-6 Si: 0-2 76Se: 0-2 23Na: 0-1

QToF Premier

20-May-2019
DG_NBoc_NFmoc_Aph_OAll 517 (4.343) Cm (514:521)

1: TOF MS ES-
6.31e+002

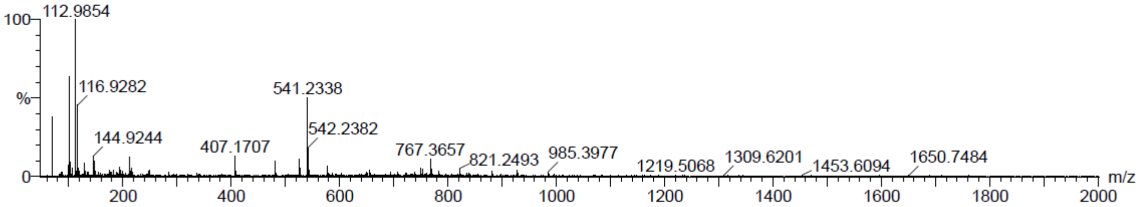

Mass	Calc. Mass	mDa	PPM	DBE	i-FIT	i-FIT (Norm)	Formula
Minimum:				-1.5			
Maximum:		3.0	5.0	100.0			
541.2338	541.2339	-0.1	-0.2	17.5	63.5	1.8	C32 H33 N2 O6
	541.2341	-0.3	-0.6	6.5	63.9	2.2	C25 H45 N2 O4 Si 76Se
	541.2343	-0.5	-0.9	16.5	70.2	8.5	C31 H37 N2 O3 Si2

Figure S2: HR-QToF(ESI-)MS analysis of **7**.

N-Trt-(NFmoc)Aph-OAll (8):

Crude N-*tert*-butoxycarbonyl-N-(9-fluorenylmethoxycarbonyl)-L-4-aminophenylalanine allyl ester, **7** (4.9 g, approx. 9.0 mmol) was dissolved in 40 mL of DCM and 10 mL of TFA were added. Then the solution was stirred at room temperature for 4 h and the solvents evaporated under reduced pressure. The crude material was taken to 50 mL of H_2O/MeCN 3:1 and freeze-dried to afford 5.0 g of the corresponding N-(9-fluorenylmethoxycarbonyl)-L-4-aminophenylalanine allyl ester trifluoroacetate salt (8.9 mmol). The product was re-dissolved in MeCN/DCM 2:1 (100 mL) and TrtCl (5 grams, 17.8 mmol) was added to the solution. The mixture was stirred at 0 ºC for 30 min and then 6 mL of DIPEA were slowly added dropwise (4 equiv., 35.6 mmol). The reaction was allowed to proceed for 2 h and formation of the products followed by TLC chromatography. Upon completion, solvents were evaporated under *vacuum* and the crude mixture taken to EtOAc (100 mL), washed with water (2 x 50 mL) and brine, dried over $MgSO_4$ and concentrated. Chromatographic purification using 15% EtOAc in hexane afforded **8** (4.3 g, 69% yield) as an off-white solid powder.

^{1}H NMR (700 MHz; $CDCl_3$) δ: 7.81 (d, J = 7.6 Hz, 2H), 7.65 (d, J = 7.5 Hz, 2H), 7.50-7.40 (m, 8H), 7.35 (t, J = 7.4 Hz, 2H), 7.25-7.22 (m, 8H), 7.20-7.16 (m, 5H), 5.57 (ddt, J = 16.6, 10.4, 6.0 Hz, 1H), 5.15-5.05 (m, 2H), 4.57 (d, J = 6.7 Hz, 2H), 4.30 (t, J = 6.7 Hz, 1H), 3.99 (ddt, J = 13.0, 5.9, 1.3 Hz, 1H), 3.85 (ddt, J = 13.0, 6.1, 1.3 Hz, 1H), 3.61 (d, J = 6.8 Hz, 2H), 2.99-2.91 (m, 1H). ^{13}C NMR (176 MHz; $CDCl_3$) δ:174.1, 153.4, 146.0, 143.8, 141.4, 136.5, 132.7, 131.9, 130.6, 128.9, 127.9, 127.9, 127.2, 126.4, 126.4, 125.0, 120.1, 71.1, 66.8, 65.3, 58.3, 47.2, 41.7. ESI(+)-LC/MS: 707.46 $[M+Na]^+$. ESI(-)-LC/MS: 729.49 $[M+CO_2H]^-$; HRMS (ESI) m/z: [M + Na]+ Calcd for C46H40N2O4Na 797.2899; Found: 797.2914

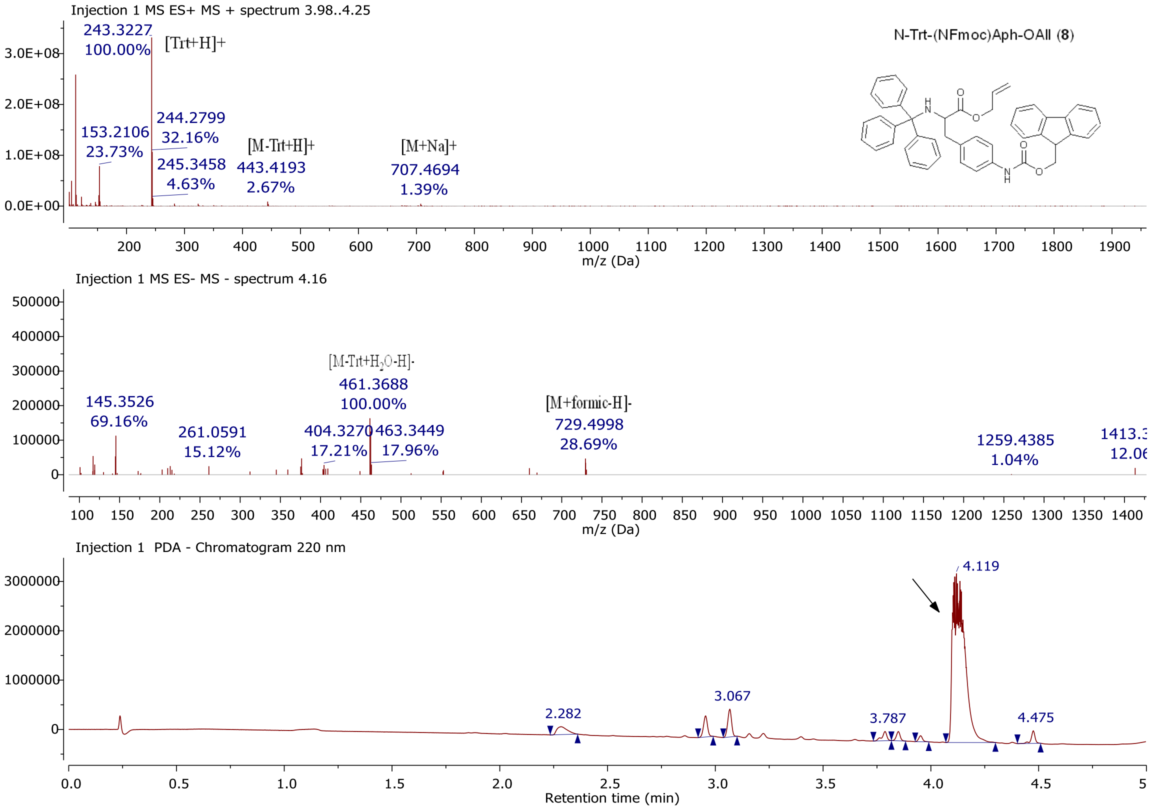

Figure S3: (ESI-/+)LC/MS trace at λ = 220 nm of crude compound **8**.

Elemental Composition Report

N-Trt-(NFmoc)Aph-OAll (**8**)

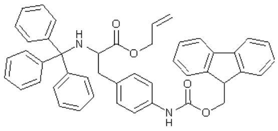

Single Mass Analysis

Tolerance = 5.0 PPM / DBE: min = -1.5, max = 100.0
Element prediction: Off
Number of isotope peaks used for i-FIT = 5

Monoisotopic Mass, Even Electron Ions
4171 formula(e) evaluated with 12 results within limits (up to 500 closest results for each mass)
Elements Used:
C: 0-50 H: 0-50 N: 0-7 O: 0-6 Si: 0-2 76Se: 0-2 23Na: 0-1

QToF Premier

20-May-2019
DG_Ntrt_NFmoc_Aph_OAll 690 (5.799) Cm (690:699)

1: TOF MS ES+
3.04e+001

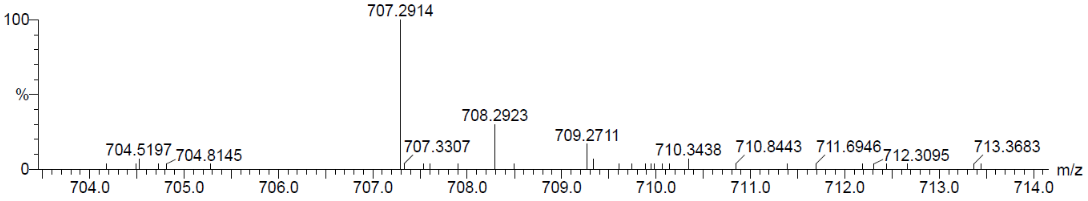

		mDa	PPM	DBE	i-FIT	i-FIT (Norm)	Formula
Minimum:				-1.5			
Maximum:		3.0	5.0	100.0			

Mass	Calc. Mass	mDa	PPM	DBE	i-FIT	i-FIT (Norm)	Formula
707.2914	707.2880	3.4	4.8	24.5	33.9	2.5	C45 H47 N2 O 76Se
	707.2886	2.8	4.0	27.5	33.8	2.5	C46 H40 N2 O4 23Na
	707.2899	1.5	2.1	32.5	33.9	2.6	C47 H36 N6 23Na
	707.2914	0.0	0.0	29.5	34.2	2.8	C47 H43 N2 O Si2
	707.2910	0.4	0.6	30.5	33.8	2.5	C48 H39 N2 O4
	707.2923	-0.9	-1.3	35.5	34.0	2.6	C49 H35 N6

Figure S4: HR-QToF(ESI+)MS analysis of **8**.

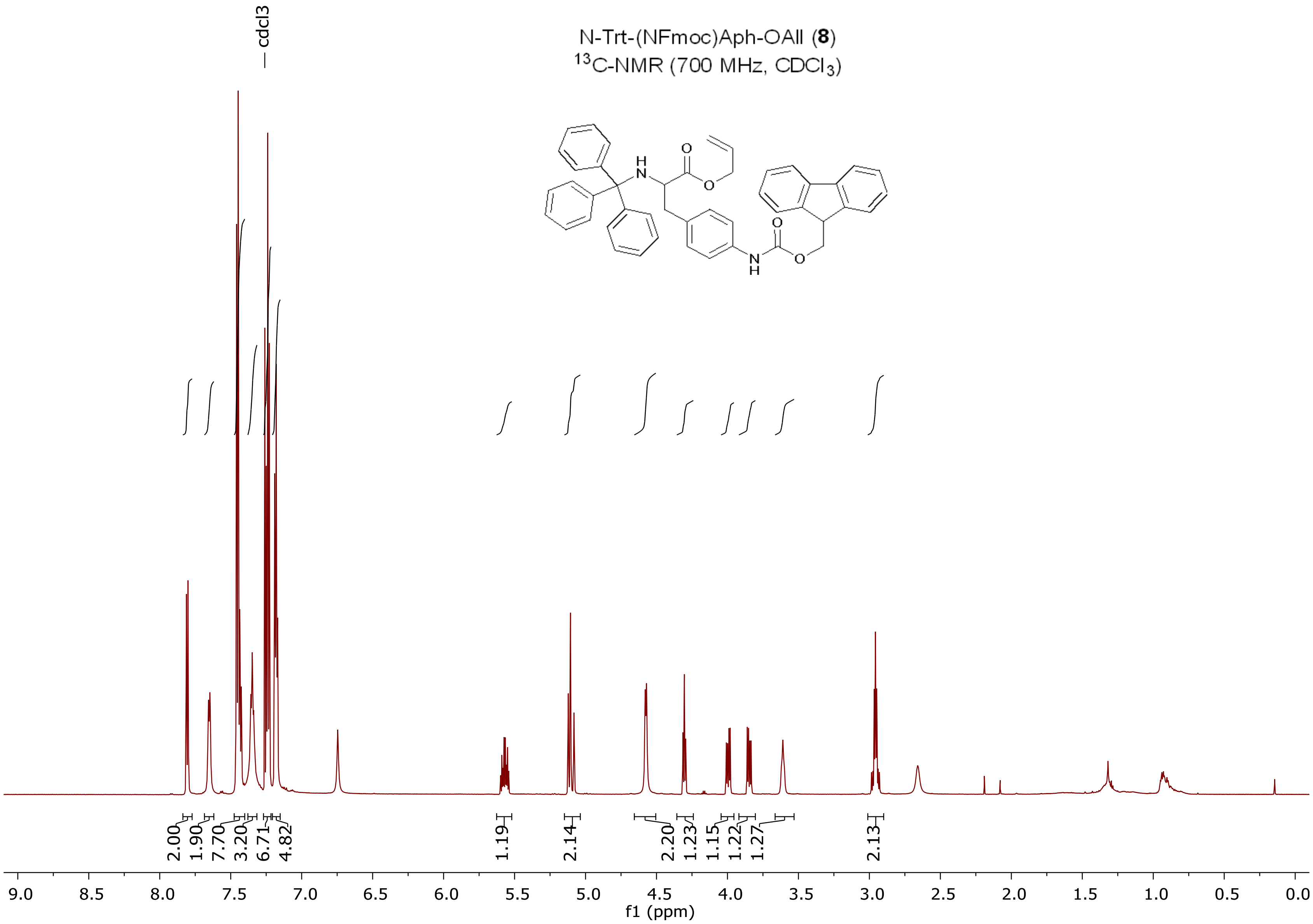

Figure S5: ^{1}H-NMR (700 MHz, $CDCl_3$) spectra of compound **8** at room temperature.

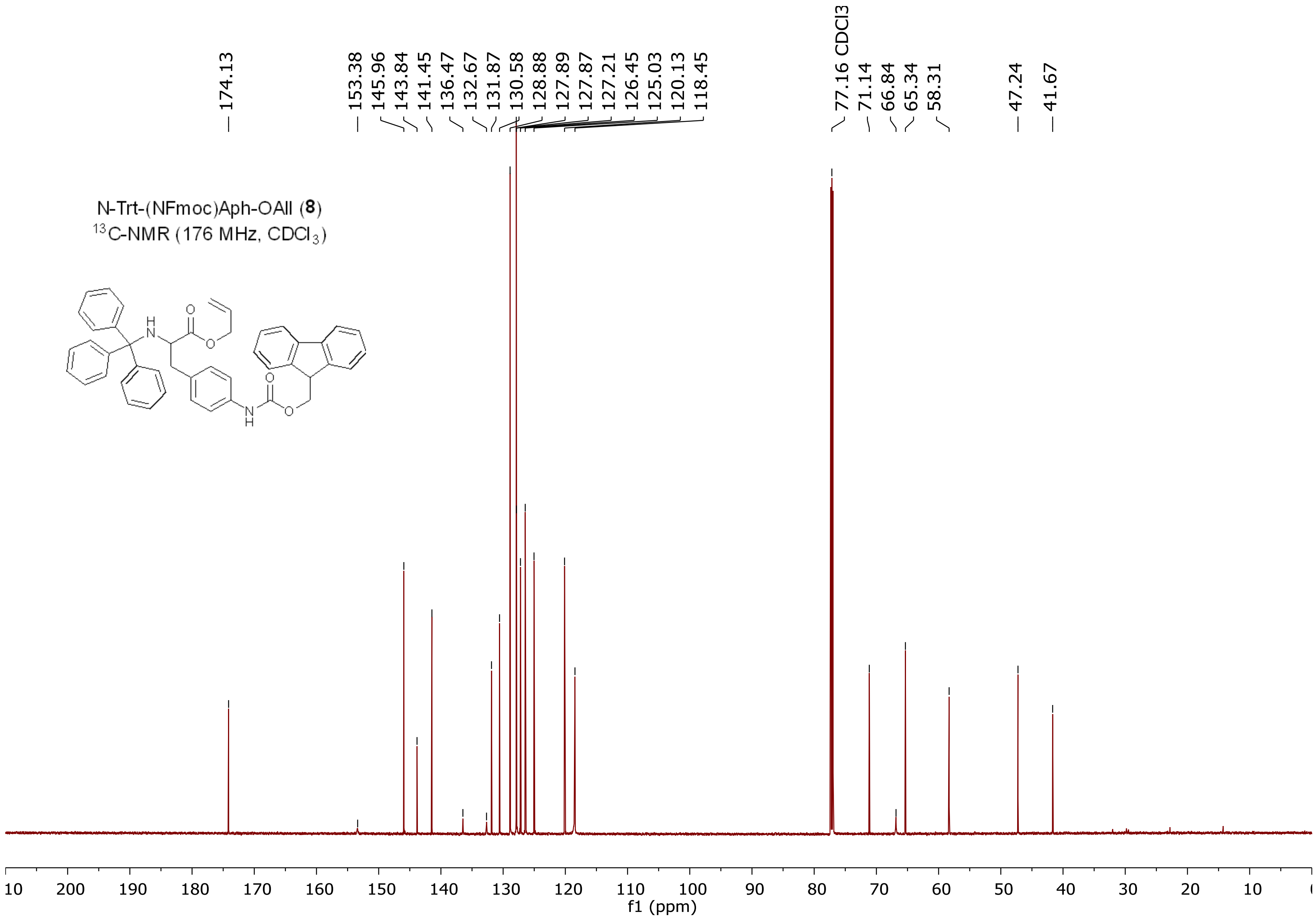

Figure S6: ^{13}C-NMR (176 MHz, $CDCl_3$) spectra of compound **8** at room temperature.

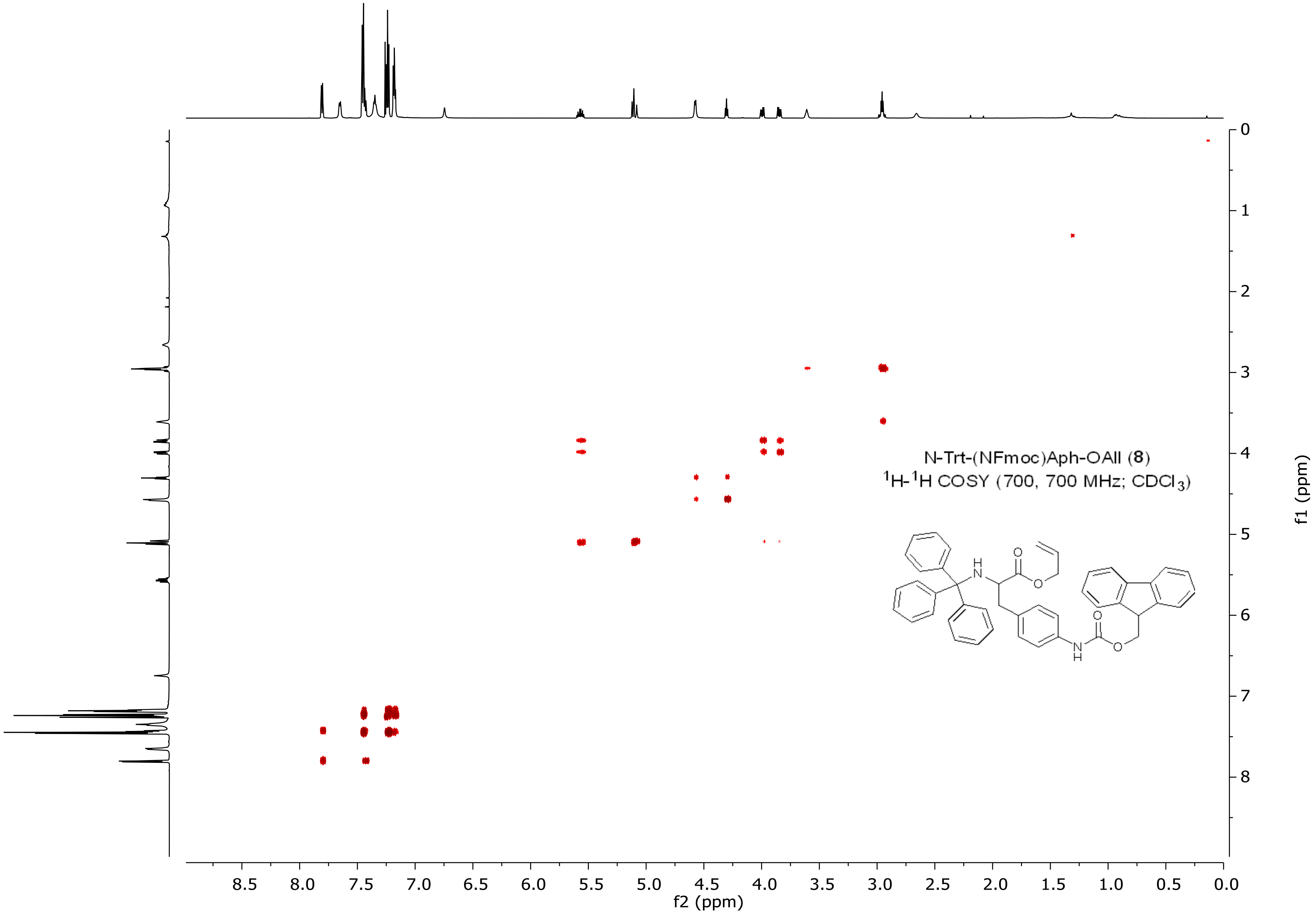

Figure S7: ^1H-^1H COSY (700 MHz, $CDCl_3$) spectra of compound **8** at room temperature.

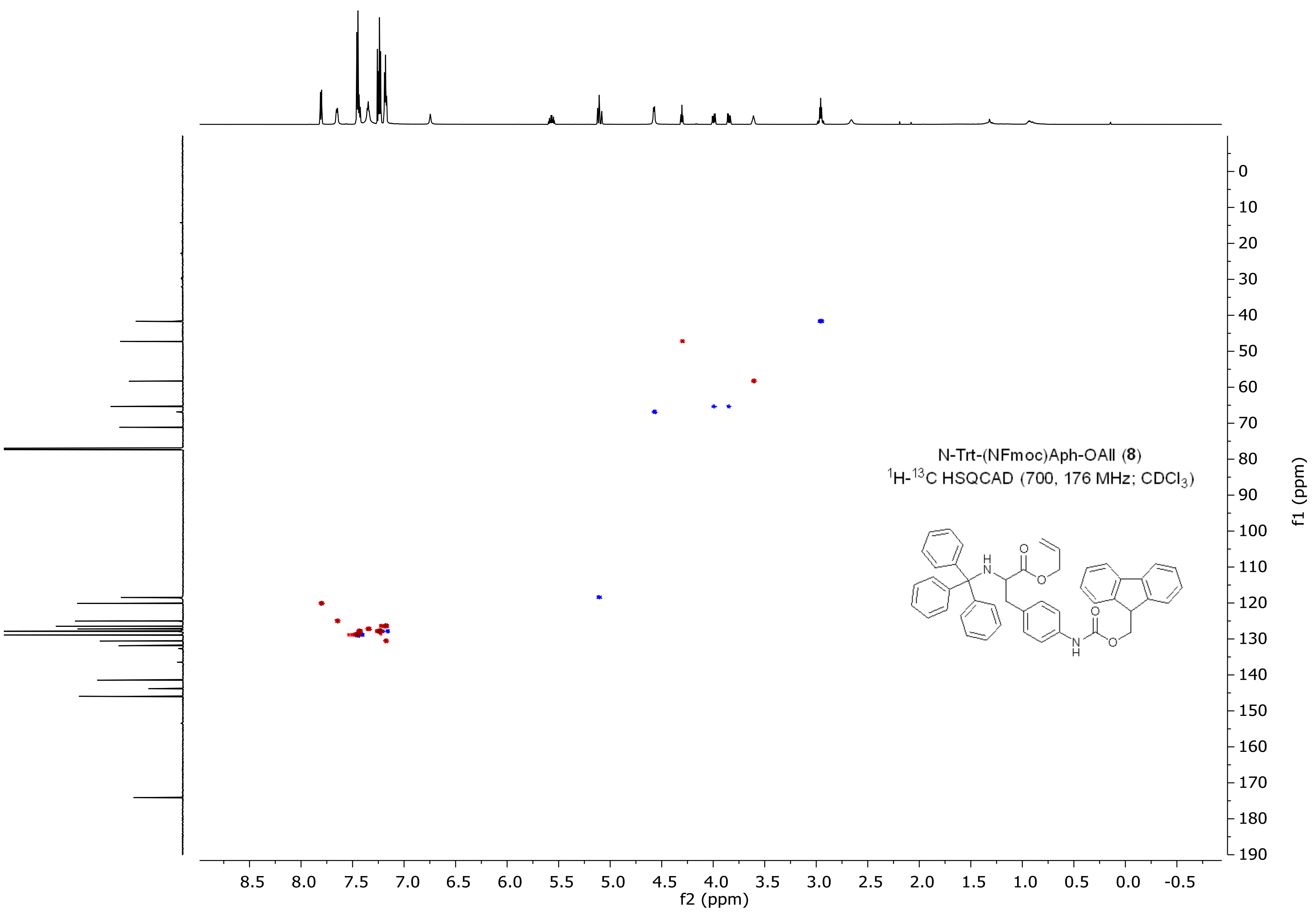

Figure S8: ^{1}H-^{13}C HSQCAD (700, 176 MHz, $CDCl_3$) spectra of compound **8** at room temperature.

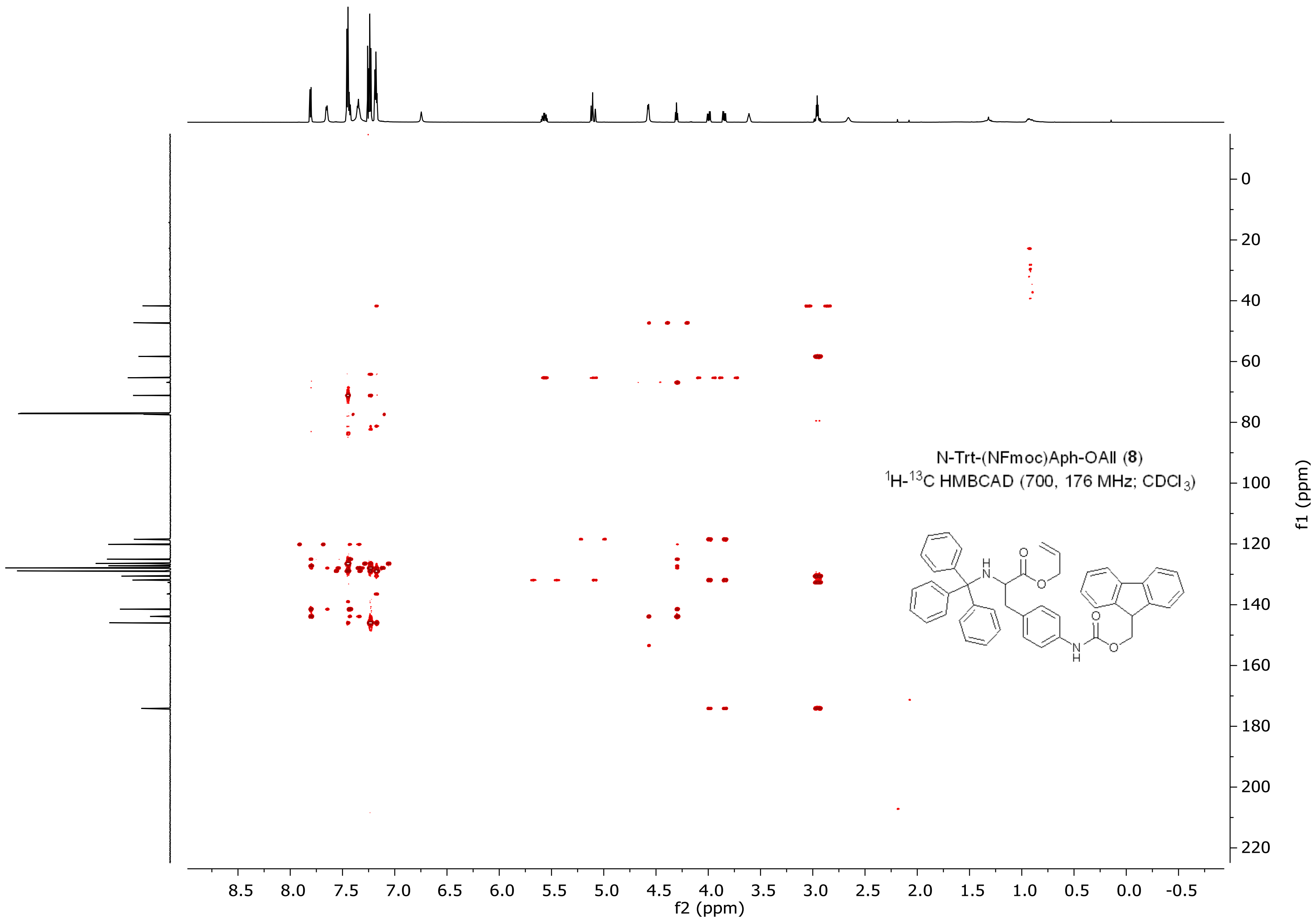

Figure S9: ^{1}H-^{13}C HMBCAD (700, 176 MHz, $CDCl_3$) spectra of compound **8** at room temperature.

Trt(NFmoc)Aph-OH (9):

A mixture of tetrakis(triphenylphosphine)palladium(0) (381 mg, 0.33 mmol) and PPh_3 (86 mg, 0.33 mmol) was stirred in DCM (15 mL) at room temperature for 5 min. This solution was then transferred via cannula to a stirred solution of N-trityl-N-(9-fluorenylmethoxycarbonyl)-L-4-aminophenylalanine allyl ester (**8**) (1.5 g, 2.2 mmol) and phenylsilane (0.54 mL, 4.4 mmol) in DCM (50 mL) at room temperature and further stirred for another 2 h. Then the reaction mixture was diluted with DCM (50mL) and was washed with water (2 x 50 mL) and brine, dried over $MgSO_4$ and the solvent evaporated under *vacuum*. 2.5 g of the crude mixture were recovered showing no traces corresponding to the OAll group of the starting material as assessed by ^{1}H-NMR, indicating quantitative removal of the carboxyl protecting group (**Figure S10**). This crude material was employed for peptide synthesis without any further purification.

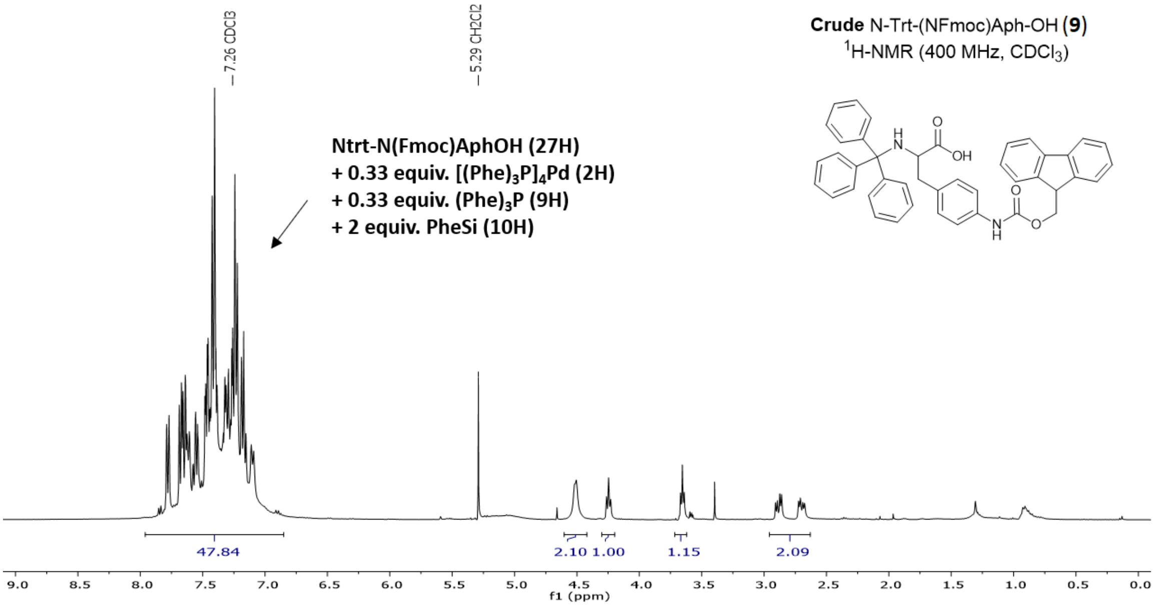

Figure S10: ^{1}H-NMR (400 MHz, $CDCl_3$) spectra of the crude reaction mixture of compound **9** at room temperature

For characterization purposes, chromatographic purification of **9** was found to be challenging due to its double acid/base labile character. Indeed, we observed complete cleavage of the N-terminus protection upon silica flash column purification. However, product purification could be accomplished by prep-TLC chromatography using plates (Analtech, silica matrix, 20 x 20 cm, 1.500 μm) that were pre-deactivated with diluted DIPEA in DCM (1% *v/v*). 100 mg of the crude mixture (~ 0.09 mmol of product) was loaded and the plate raised for three consecutive times with EtOAc:DIPEA 9.9: 0.1 *v/v*. UV absorbing bands corresponding to single products were scratched from the plate, extracted with EtOAc/MeOH 95:5 *v/v* and then filtered. Concentration of the organic layer under *vacuum* afforded 52 mg of compound **9** (DIPEA solvate) as a colourless syrup.

^{1}H NMR (700 MHz; $CDCl_3$) δ: 7.77 (d, J = 7.6 Hz, 2H), 7.61 (d, J = 7.6 Hz, 2H), 7.44-7.38 (m, 8H), 7.31 (t, J = 7.4 Hz, 2H), 7.23-7.17 (m, 8H), 7.16-7.10 (m, 5H), 4.51 (d, J = 6.8 Hz, 2H), 4.26 (t, J = 6.8 Hz, 2H), 3.53 (t, J = 5.6 Hz, 1H), 2.78 (dd, J =13.5, 5.4 Hz, 1H), 2.48 (dd, J =13.5, 5.8 Hz, 1H); ^{13}C NMR (176 MHz; $CDCl_3$) δ: 177.4, 157.6, 146.5, 143.9, 141.5, 135.8, 133.9, 130.8, 129.1, 127.9, 127.9, 127.2, 126.4, 126.4, 125.1, 120.7, 71.6, 66.9, 58.7, 47.3, 40.38. HRMS (ESI) m/z: $[M - H]^-$ Calcd for C43H35N2O4 643.2597; Found: 643.2623

Single Mass Analysis

Tolerance = 5.0 PPM / DBE: min = -1.5, max = 100.0
Element prediction: Off
Number of isotope peaks used for i-FIT = 5

N-Trt-(NFmoc)Aph-OH (**9**)

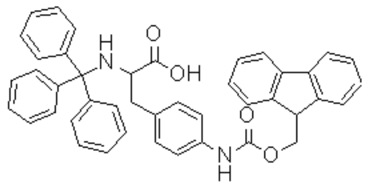

Monoisotopic Mass, Even Electron Ions
172 formula(e) evaluated with 2 results within limits (up to 500 closest results for each mass)
Elements Used:
C: 0-50 H: 0-50 N: 0-5 O: 0-6

QToF Premier

DG_NtrtNFmoc_Aph_OH 632 (5.313) Cm (619:642)

1: TOF MS ES-
2.24e+003

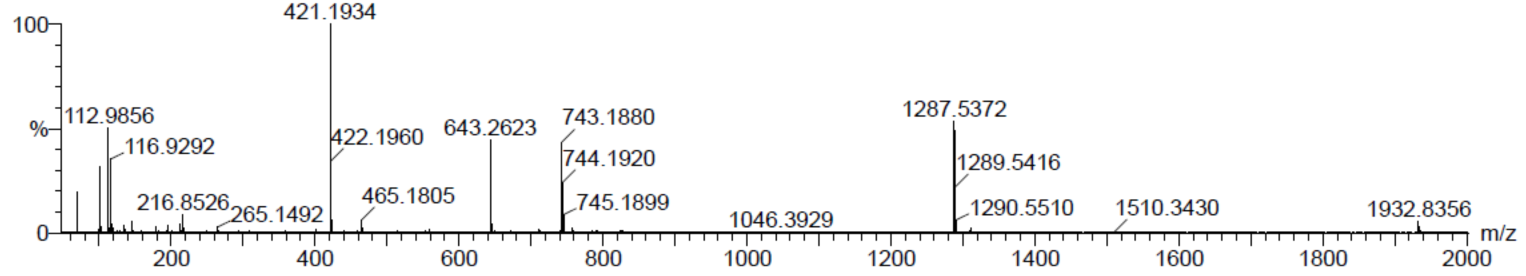

Minimum:				-1.5			
Maximum:		3.0	5.0	100.0			
Mass	Calc. Mass	mDa	PPM	DBE	i-FIT	i-FIT (Norm)	Formula
643.2623	643.2637	-1.4	-2.2	31.5	141.5	2.4	C48 H35 O2
	643.2597	2.6	4.0	27.5	139.2	0.1	C43 H35 N2 O4

Figure S11: HR-QToF(ESI-)MS analysis of **9**.

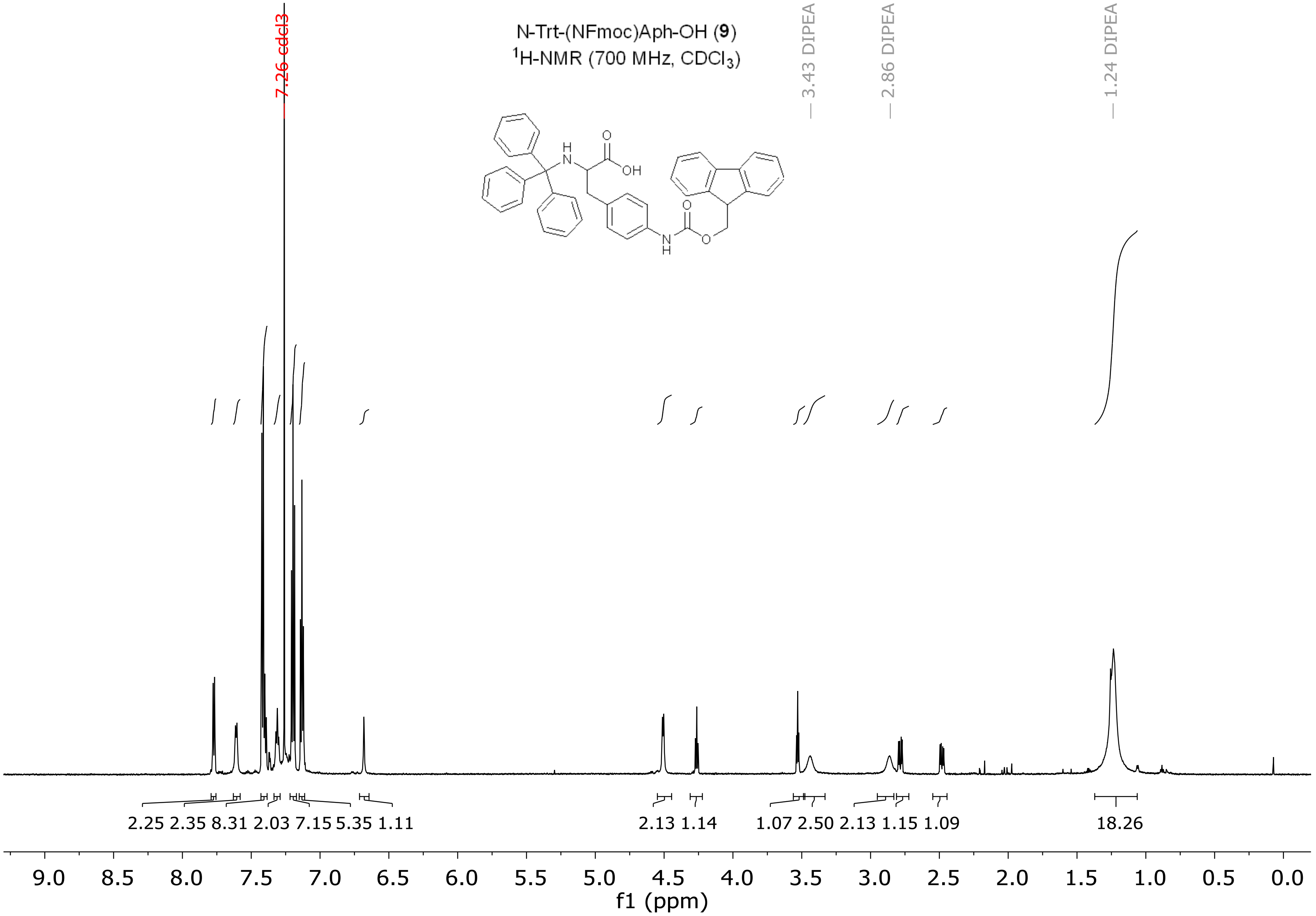

Figure S12: ^{1}H NMR (700 MHz, $CDCl_3$) spectra of compound **9** at room temperature.

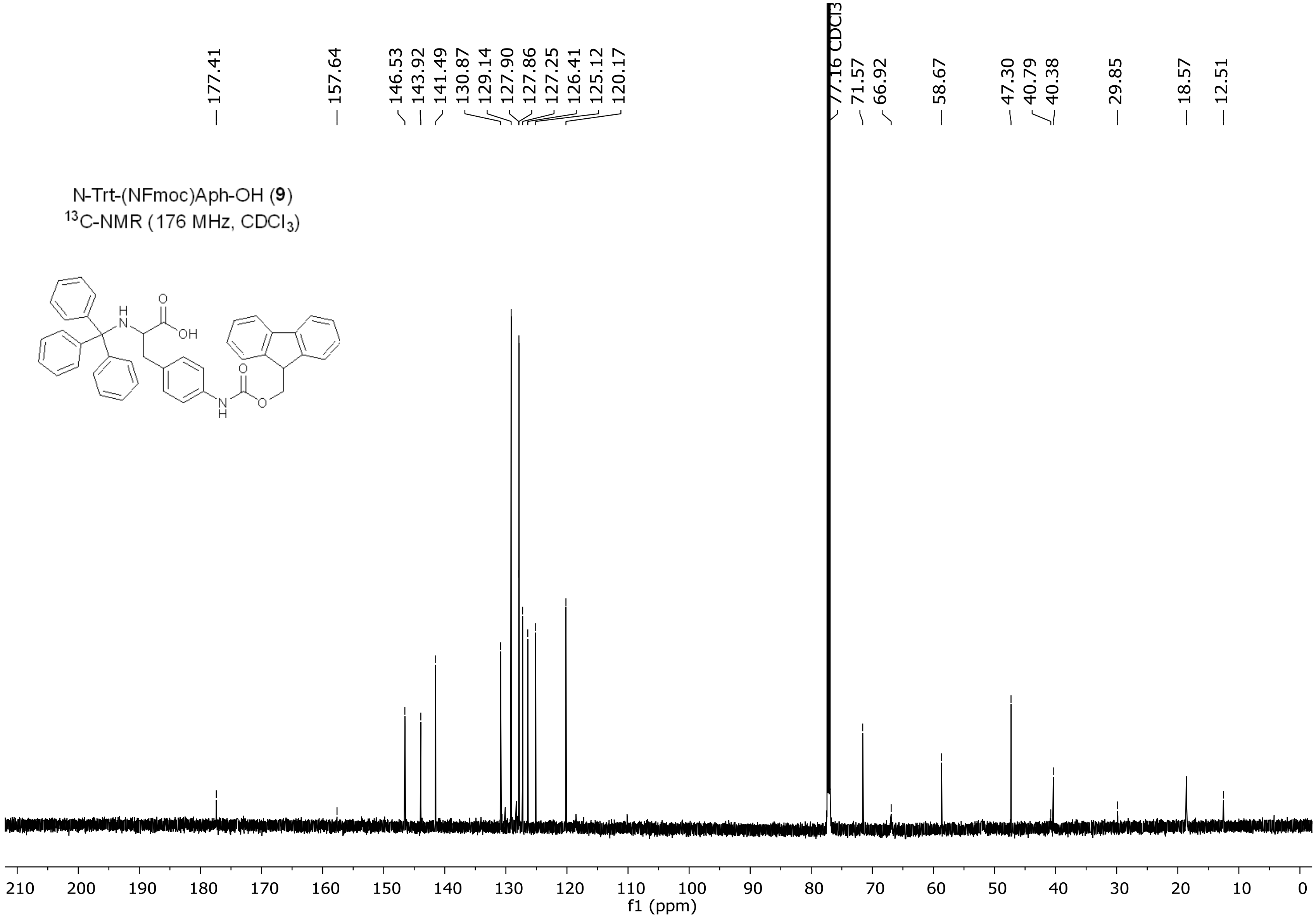

Figure S13: ^{13}C NMR (176 MHz, $CDCl_3$) spectra of compound **9** at room temperature.

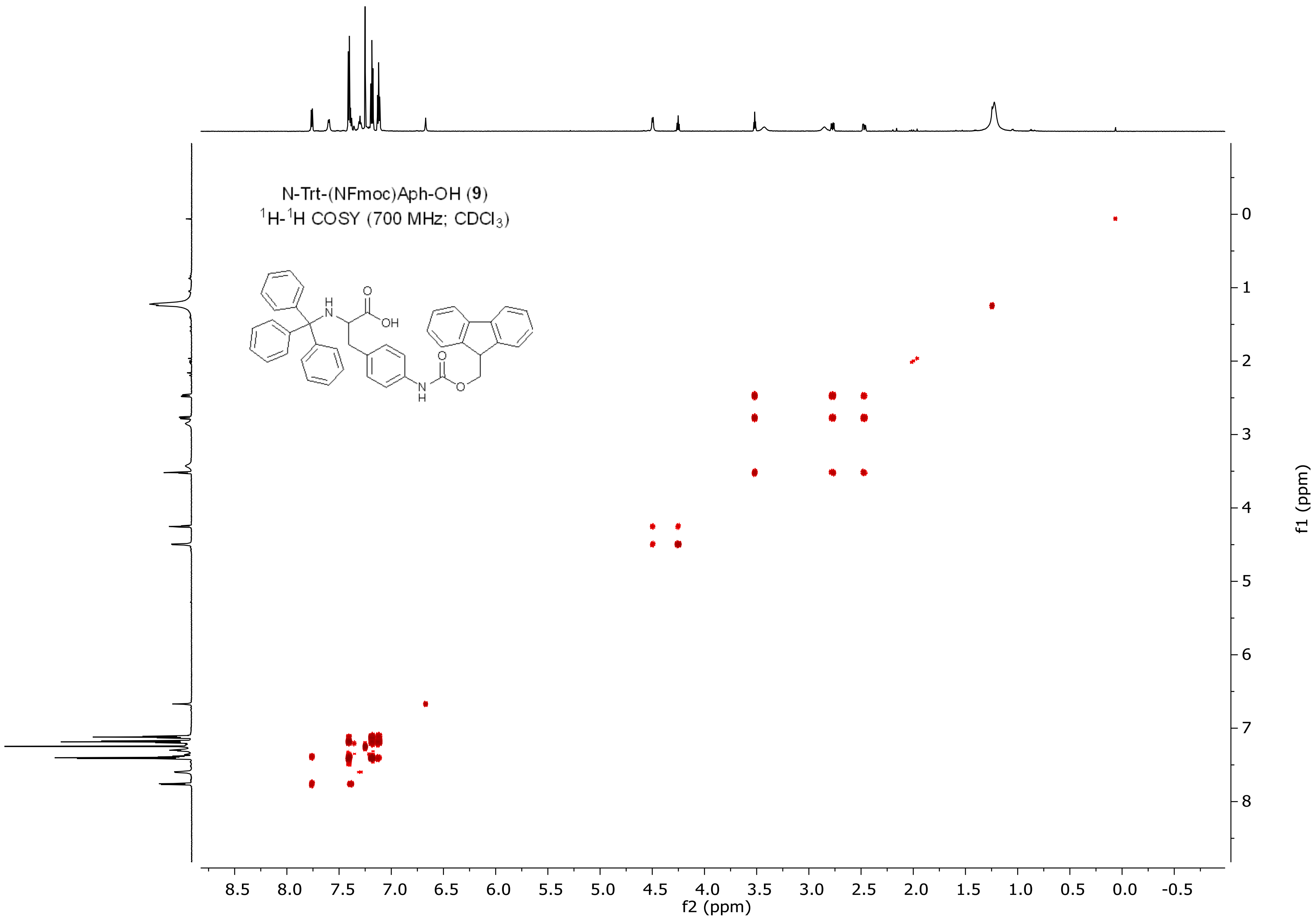

Figure S14: ^{1}H-^{1}H COSY (700 MHz, $CDCl_3$) spectra of compound **9** at room temperature.

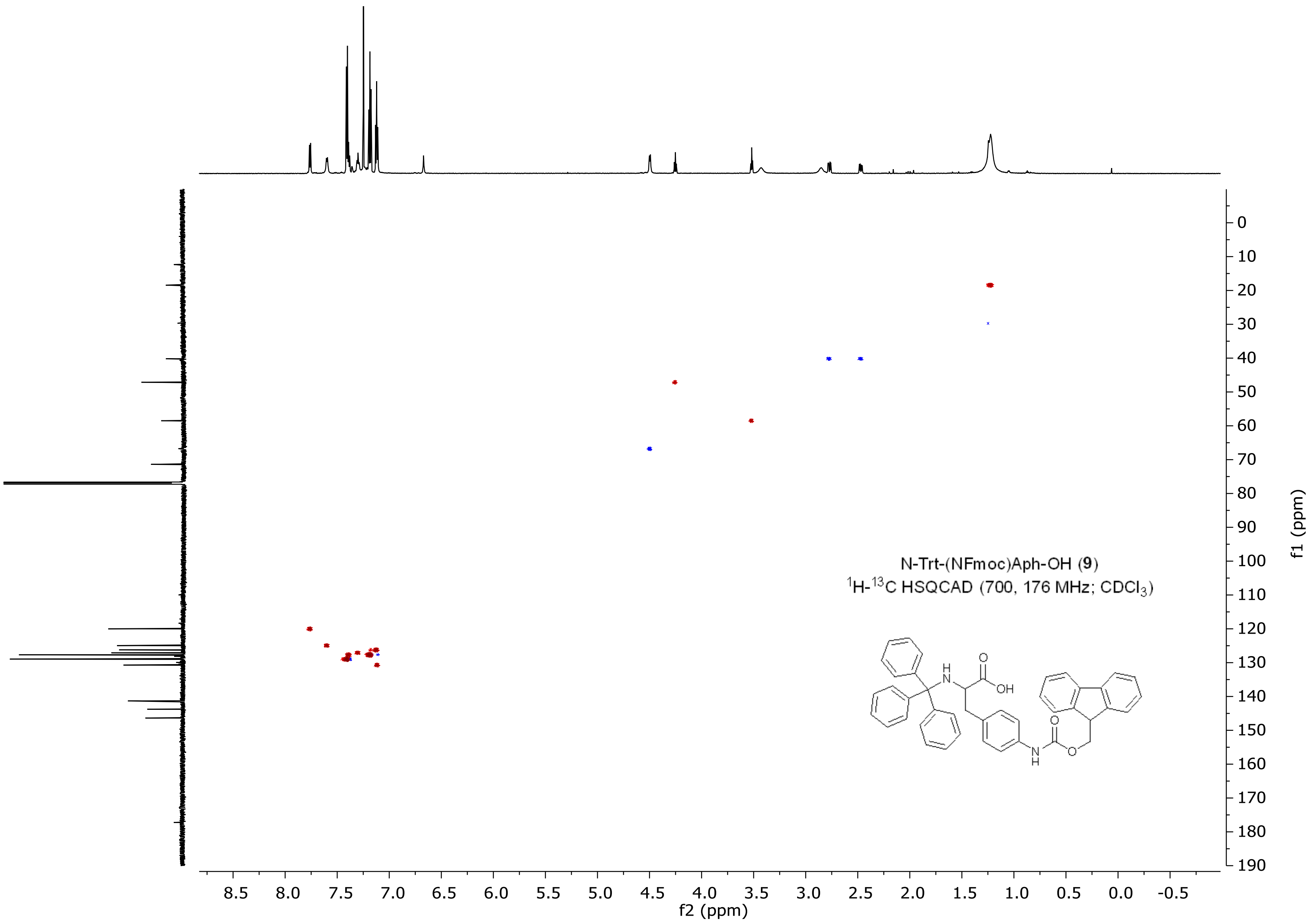

Figure S15: ^{1}H-^{13}C HSQCAD (700, 176 MHz, $CDCl_3$) spectra of compound **9** at room temperature.

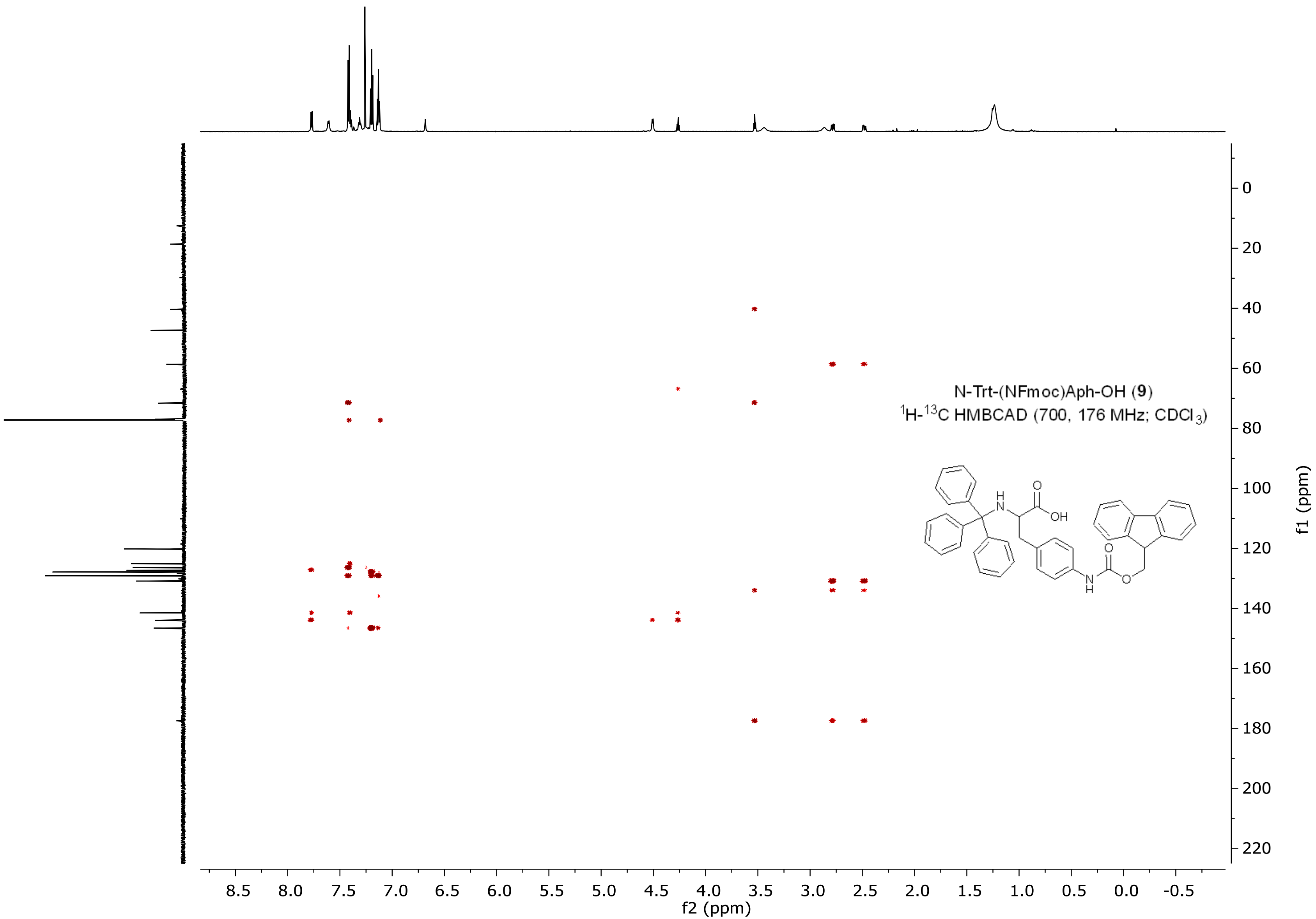

Figure S16: ^{1}H-^{13}C HMBCAD (700, 176 MHz, $CDCl_3$) spectra of compound **9** at room temperature.

Solid-Phase Peptide Synthesis

Solid-Phase Peptide Synthesis of linear peptide 5 and cyclic peptide 6:

Linear peptide sequence **5** was synthesized manually using standard Fmoc solid-phase peptide synthesis protocols with the reaction vessel placed in an orbital shaker platform (400 r.p.m). *Fmoc-Gln(Dmab)-OH, Fmoc-Pro-OH, Fmoc-D-Ala-OH, Fmoc-Glu(tBu)-OH, Fmoc-D-Thr(tBu)-OH, Fmoc-L-Ile-OH, Fmoc-D-Tyr-OH and Boc-L-(NFmoc)Aph-OH were used as amino acid derivatives.* 50 mg of Rink-amide resin (NovaBiochem; 200 mesh; 0.81 mmol/g) was washed twice in DMF (2 mL) and swollen in the same solvent for 30 min. Then the first amino acid was coupled by reacting 0.2 mmol of Fmoc-Glu-ODmab (5 molar equiv.) in the presence of *of N,-diisopropylcarbodiimide (DIC;* 6 molar equiv.*) and N*-Hydroxybenzotriazole (*HOBt;* 6 molar equiv.*)* for 3 h, followed by extensive washes with DMF (4X, DMF, 2 mL). Resin loaded with Fmoc-Glu-ODmab was incubated twice with 2 mL of 20% *v/v* piperidine/DMF for 15 min at room temperature to accomplish the Fmoc group deprotection, which was followed by extensive washes with DMF (4X, DMF, 2 mL). All subsequent amino acid couplings and Fmoc deprotections steps were performed following the same guideline until the desired linear sequence was obtained, except for *Boc-L-(NFmoc)Aph-OH*, that was incorporated by employing 3 molar equivalents of amino acid in the presence of 4 molar equiv. of activating reagents. The completion of each coupling was monitored by a Kaiser test.[1] Removal of the Dmab protecting group at the C-terminus was achieved by incubating the peptidyl-resin with 1 mL of NH_2NH_2/DMF (5% *v/v*) for 5 min. This step was repeated 5 times consecutively. Then, the resin was washed extensively (4X, DMF, 2 mL) and incubated for 1 h in 3mL of NaOH 5mM (H_2O:MeOH 1:1 *v/v*). This step was done to ensure Dmab aminobenzyl moiety elimination.[2] Once completed, the beads were carefully washed with DMF and DCM (3 x 10 ml) and a sample of the peptidyl-resin was cleaved from the resin by treatment with a mixture of TFA/TIPS/H_2O 95:5:5% *v/v* (0.2 mL) at room temperature for 2 h. LC/MS analysis of the crude material, shown next, proved the quantitative presence of the corresponding C-terminal carboxylic acid prior to peptide cyclization (see also **Figure 2** in the main text).

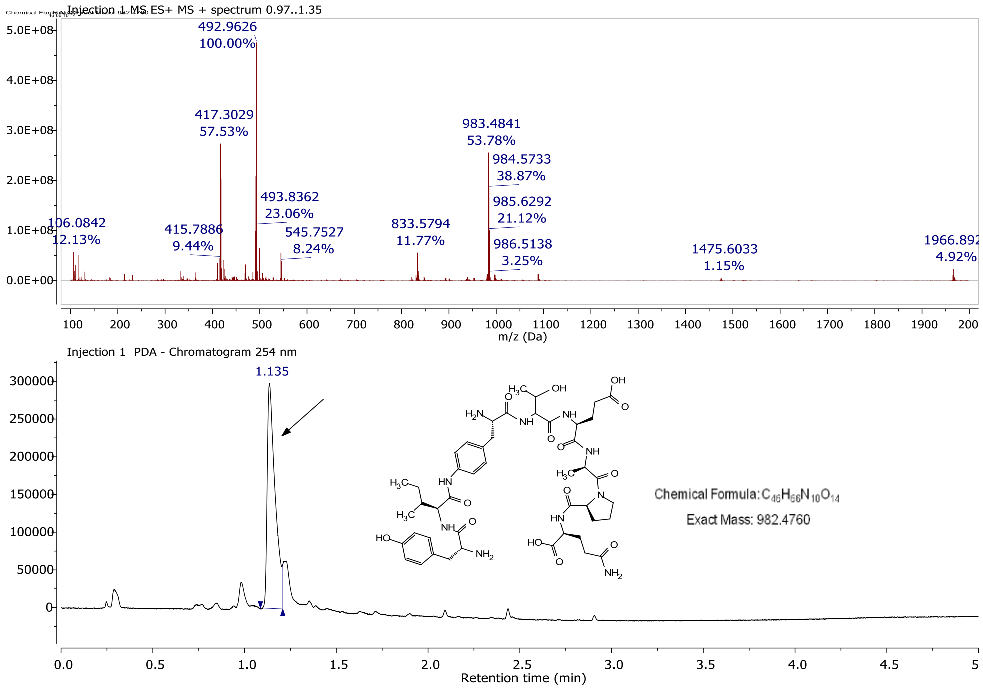

Figure S17: (ESI+)LC/MS trace at λ = 254 nm of crude compound **5**, corroborating the full deprotection of the C-terminus acid functionality after hydrazine Dmab group removal.

Once verified the presence of linear peptide **5**, the resin was re-swollen in DMF (4 mL, 1h) and reacted in the presence of DIC and HOBt overnight (1.5 molar equiv.; 1.5 mL DMF). Upon completion, the resin was washed with DMF and the treatment repeated for a second time using a fresh DIC/HOBt solution for 2 h (3 molar equiv. each, 1.5 mL). Completion of the reaction was checked with the Kaiser test, and the resin washed with DMF and DCM (3 x 10 ml) as previously. The resulting peptidyl resin was then treated with trifluoroacetic acid (TFA/H_2O/triisopropylsilane (TIPS) (95:2.5:2.5 *v/v*; 1 mL) for 2 h at room temperature and the resulting crude peptide precipitated over diethyl ether and decanted to give a white solid that was taken up in H_2O/CH_3CN (1:1), lyophilized, and analysed by LC/(ESI+)MS spectrometry. As shown below, sample analysis at λ = 254 nm, characteristic of the peptide Tyrosine residues, showed almost complete conversion of the linear peptide to the expected cyclic product **6** (also shown in **Figure 2** of the main text).

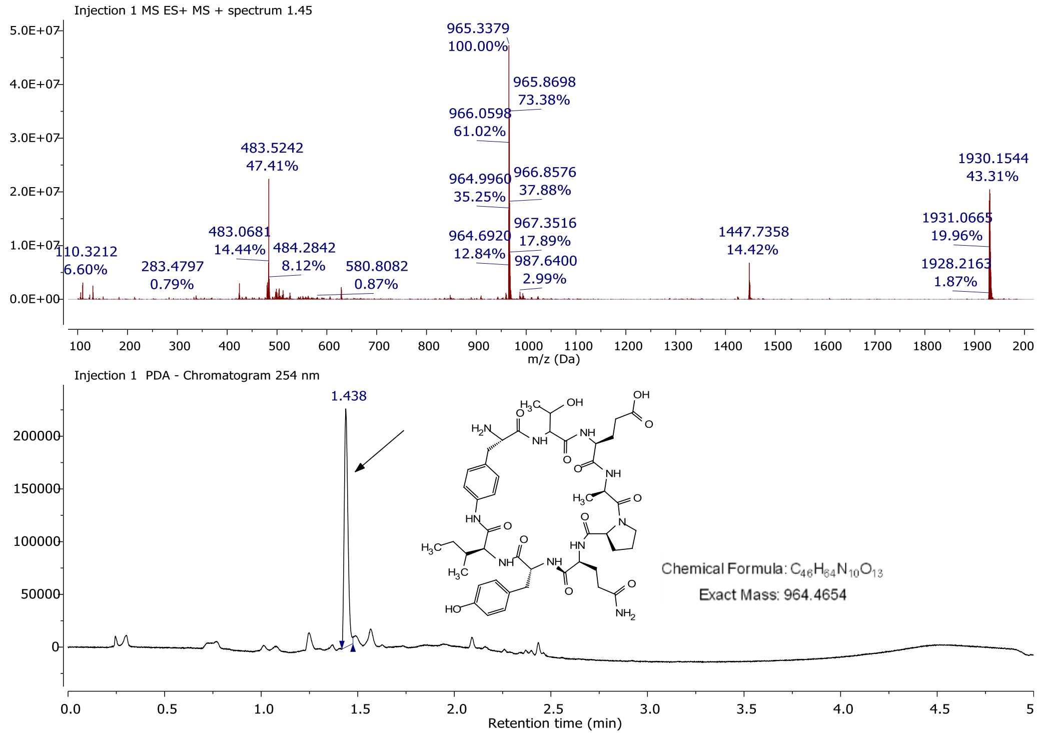

Figure S18: (ESI+)LC/MS trace at λ = 254 nm of peptide **5** after cyclization, showing the formation of the expected cyclic product **6**.

Solid-Phase Peptide Synthesis cyclic lipopeptides 17A-17D:

To obtain lipopeptides **17A**-**17D**, the same experimental procedures as described for **6** were used, employing in each case the corresponding desired Threonine derivative: Fmoc-D-Thr-OH (compound **17A**), Fmoc-L-Thr-OH (compound **17B**), Fmoc-D-AlloThr-OH (compound **17C**) and Fmoc-L-AlloThr-OH (compound **17D**). Then, incorporation of Trt-L-(NFmoc)Aph-OH, in replacement of the Boc-L-(NFmoc)Aph-OH derivative previously employed was achieved by directly coupling a portion of the crude material containing 3 molar equivalents of amino acid (135 mg; ~0.12 mmol) in the presence of DIC and HOBt (4 equiv. each). Then the peptidyl resin was extensively washed with DMF (4X, 2 mL), DCM (4X, 2 mL) and DMF for a second time (4X, 2 mL) and the synthesis resumed as described above. Following peptide cyclization, the trityl group was removed by treating the resin 5 consecutive times with a diluted solution of TFA/TIPS in DCM (0.2/1% *v/v*, 2 mL, 1 min). Then the resin was neutralized in DIPEA/DMF (5 *v/v* %, 2 mL, 1 h) and Fmoc-D-Orn(Boc)-OH (2.5 mol equiv.) and Fmoc-L-Glu(*t*Bu)-OH (5 mol equiv.) sequentially

coupled and the final N-terminus Fmoc group deprotected (20% piperidine/DMF *v/v*). The peptidyl resin was then washed repeated times in DCM (4X, 2 mL, 5 min) and the final lipidation of the product accomplished by using palmitoyl chloride (5 mol equiv., 0.2 mmol) in the presence of DIPEA (10 mol equiv., 0.4 mmol) for 3 h. Upon completion, the resin was washed with DCM (6X, 2 mL, 2 min) and air dried. The resulting peptides were cleaved from the resin by treatment with trifluoroacetic acid (TFA/H_2O/TIS 95:2.5:2.5 *v/v*; 1 mL) for 2 h at room temperature and the crude materials precipitated in Et_2O to afford yellowish solid precipitates that were taken up in H_2O/CH_3CN (1:1), lyophilized, and analysed by analytical HPLC (see following Figures **S19-S25** and **Figure 3** in the main text). Following analysis of the crude materials, peptides **17A**-**17D** were purified by RP-HPLC to yield the corresponding cyclic lipopeptides in purities ≥ 98%, as confirmed by analytical HPLC (λ = 220 nm) and HR QToF-LC/(ESI+)MS analysis. Characterization data for each compound is summarized in following **Table S1**.

Table S1. Characterization data of cyclic lipo-peptides 17A-17D.

Peptide	Sequence	Analytical HPLC		Yields		Acc. Mass spectrometry	
		**Retention time (min)**	**Purity* (%)**	**Isolated mass (mg)**	**Yield (%)**	**Calc. Mass (Da) $C_{72}H_{111}N_{13}O_{18}$**	**Obs. Mass (Da)**
**17A**	$C_{15}H_{31}$CO-L-Glu-D-Orn-**L-Aph***-D-Thr-L-Glu-D-Ala-L-Pro-L-Gln-D-Tyr-**L-Ile***	32.9	>98%	13.9	24	$[M+2H]^{2+}$, 723.9163	723.9147
**17B**	$C_{15}H_{31}$CO-L-Glu-D-Orn-**L-Aph***-L-Thr-L-Glu-D-Ala-L-Pro-L-Gln-D-Tyr-**L-Ile***	32.3	>98%	17.9	31	$[M+2H]^{2+}$, 723.9163	723.9166
**17C**	$C_{15}H_{31}$CO-L-Glu-D-Orn-**L-Aph***-D-AlloThr-L-Glu-D-Ala-L-Pro-L-Gln-D-Tyr-**L-Ile***	33.3	>98%	23.7	41	$[M+2H]^{2+}$, 723.9163	723.9114
**17D**	$C_{15}H_{31}$CO-L-Glu-D-Orn-L-Aph*-L-AlloThr-L-Glu-D-Ala-L-Pro-L-Gln-D-Tyr-L-Ile*	32.1	>98%	16.7	29	$[M+2H]^{2+}$, 723.9163	723.9127

**Denotes the position of the amide-bond bridge*

*No other peaks visible in the HPLC traces.

Cyclic lipopeptide 17A:

Cyclic lipopeptide **17A** was synthetized in 46% purity. From the crude material, 13.9 mg of cyclic lipodepsipeptide **17A** was isolated in purity >98% (24% yield). HPLC (λ = 220 nm) tR = 32.9 min. HR-QToF (ESI) m/z: [M+2H]2+ Calcd for C72H111N13O18 723.9163; Found 723.9147

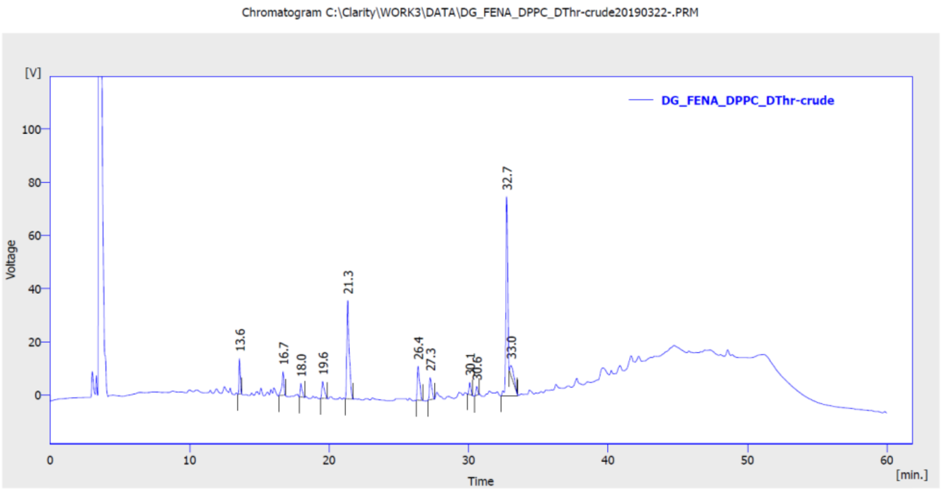

Result Table (Uncal - DG_FENA_DPPC_DThr-crude)

	Reten. Time [min]	Area [V.s]	Height [V]	Area [%]	Height [%]	W05 [min]	Compound Name
1	13.580	80.999	13.336	3.9	7.5	0.10	
2	16.703	85.863	8.980	4.2	5.0	0.13	
3	17.980	50.260	5.107	2.4	2.9	0.15	
4	19.550	75.139	6.255	3.6	3.5	0.19	
5	21.337	443.429	36.965	21.5	20.8	0.18	
6	26.387	136.898	12.738	6.6	7.2	0.18	
7	27.257	96.113	8.258	4.7	4.6	0.20	
8	30.090	42.230	4.600	2.0	2.6	0.15	
9	30.587	28.041	3.332	1.4	1.9	0.14	
10	32.737	943.263	74.830	45.7	42.0	0.16	
11	33.043	81.599	3.704	4.0	2.1	0.26	
	Total	2063.836	178.105	100.0	100.0		

Figure S19: Analytical HPLC trace at λ = 220 nm of crude peptide **17A**.

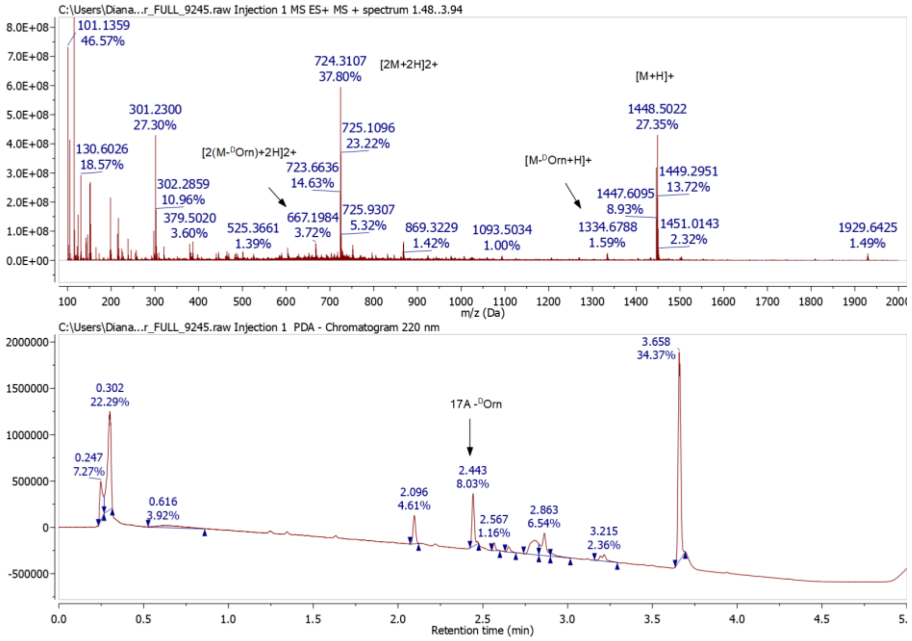

Figure S20: (ESI+)LC/MS trace of peptide crude **17A** (λ= 220 nm)**,** identifying the mass of the main by-product found as a [Dorn] depleted cyclic sequence.

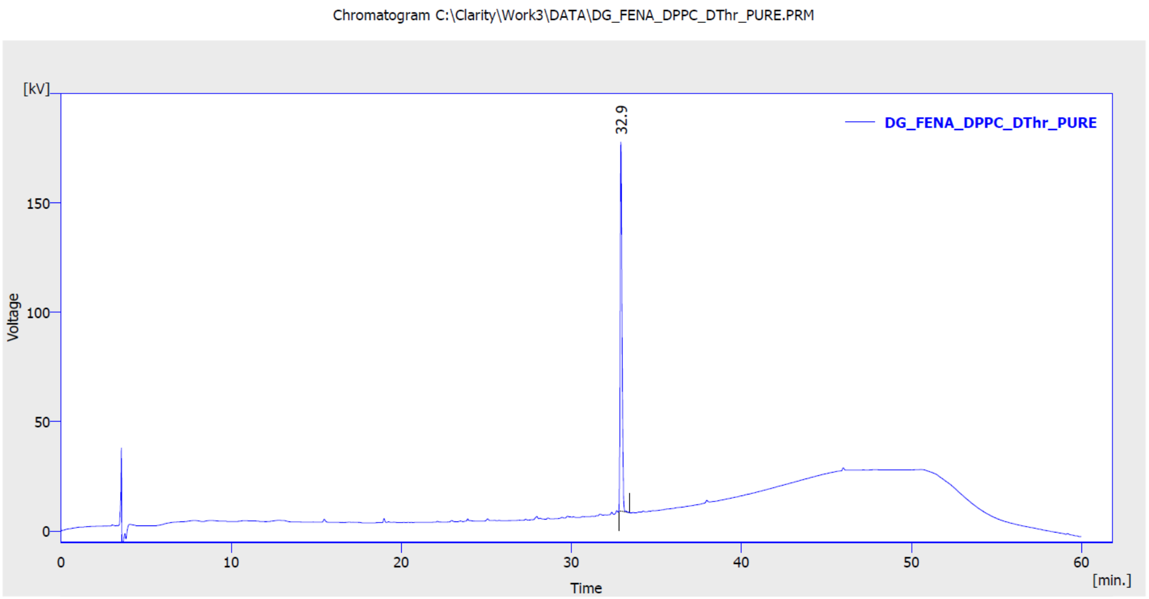

Figure S21: Analytical HPLC trace at λ = 220 nm of purified peptide **17A**.

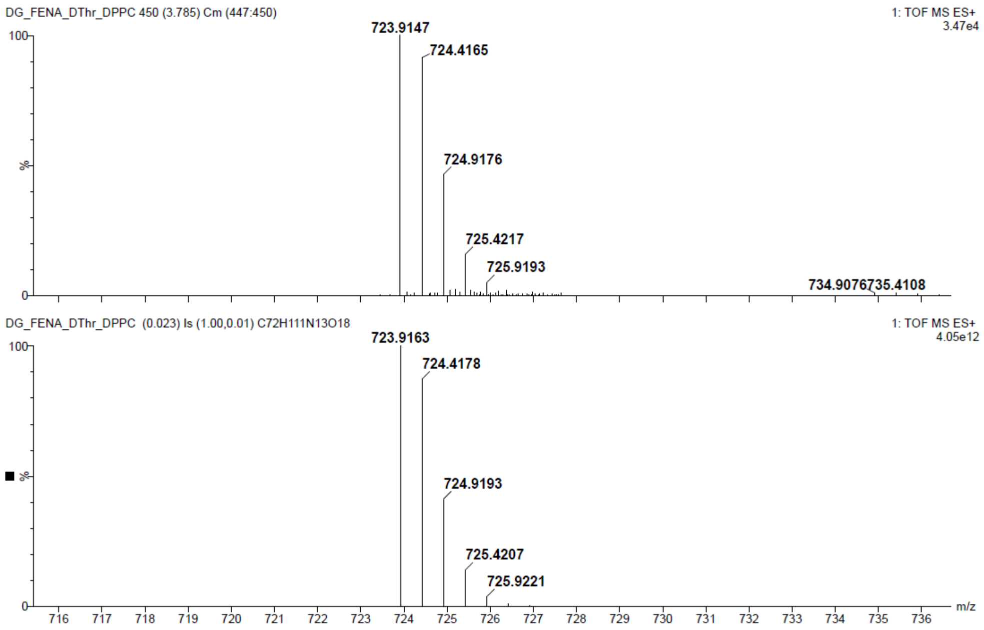

Figure S22: HR-QToF(ESI+)MS analysis of purified peptide **17A**, confirming its identity. Lower panel: calculated mass; Upper panel: experimental mass.

Cyclic lipopeptide 17B:

Cyclic lipopeptide **17B** was synthetized in 58% purity. From the crude material, 17.9 mg of cyclic lipodepsipeptide **17B** was isolated in purity >98% (31% yield). HPLC (λ = 220 nm) tR = 32.3 min. HR-QToF (ESI) m/z: [M+2H]2+ Calcd for C72H111N13O18 723.9163; Found 723.9166

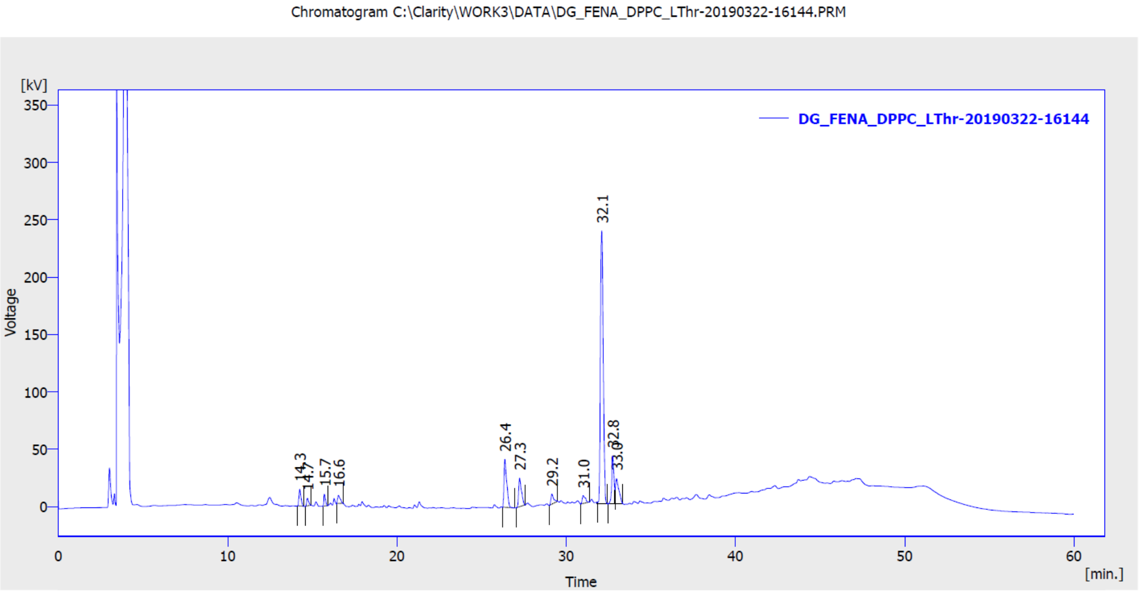

Result Table (Uncal - DG_FENA_DPPC_LThr-20190322-16144)

	Reten. Time [min]	Area [kV.s]	Height [kV]	Area [%]	Height [%]	W05 [min]	Compound Name
1	14.267	122.126	14.761	2.6	3.5	0.13	
2	14.720	53.651	6.734	1.2	1.6	0.13	
3	15.727	72.844	10.436	1.6	2.5	0.12	
4	16.557	74.510	7.076	1.6	1.7	0.19	
5	26.387	453.454	41.952	9.8	9.9	0.17	
6	27.253	290.702	25.029	6.3	5.9	0.19	
7	29.163	95.441	8.860	2.1	2.1	0.17	
8	31.010	85.747	6.812	1.8	1.6	0.24	
9	32.113	2690.410	238.003	58.0	56.2	0.18	
10	32.753	395.801	41.706	8.5	9.9	0.15	
11	32.977	307.755	21.968	6.6	5.2	0.25	
	Total	4642.441	423.337	100.0	100.0		

Figure S23: Analytical HPLC trace at λ = 220 nm of crude peptide **17B**.

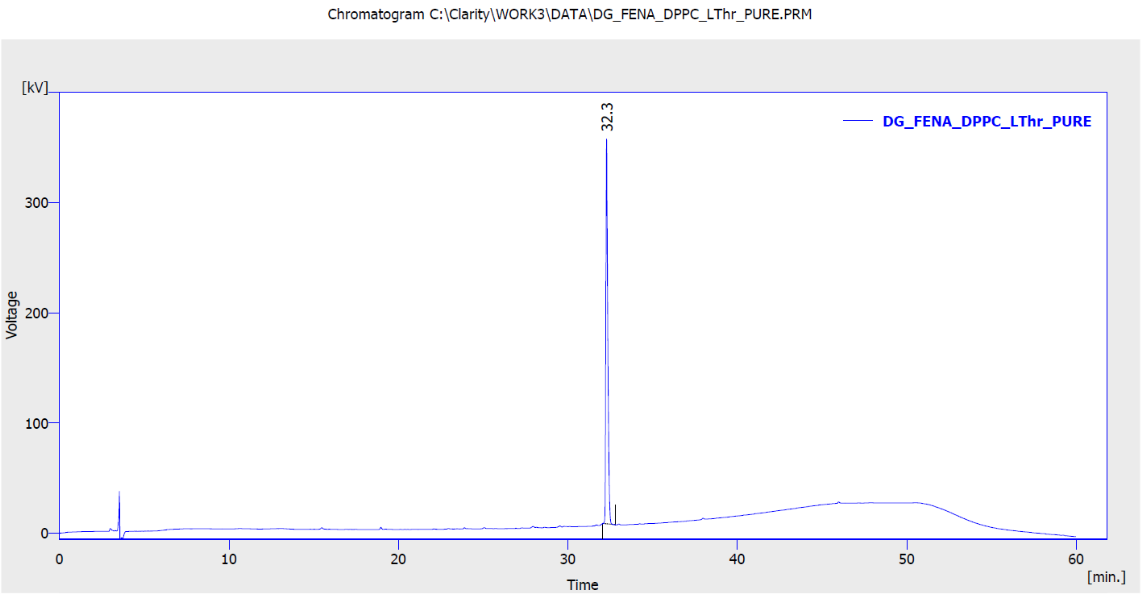

Figure S24: Analytical HPLC trace at λ = 220 nm of purified peptide **17B**.

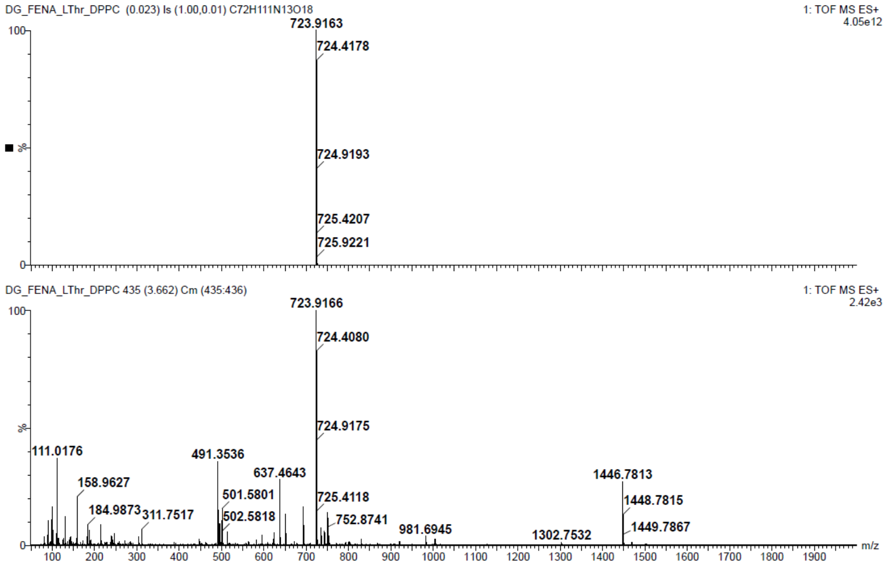

Figure S25: HR-QToF(ESI+)MS analysis of purified peptide **17B**, confirming its identity. Upper panel: calculated mass; lower panel: experimental mass.

Cyclic lipopeptide 17C:

Cyclic lipopeptide **17C** was synthetized in 71% purity. From the crude material, 23.7 mg of cyclic lipodepsipeptide **17C** was isolated in purity >98% (41% yield). HPLC (λ = 220 nm) tR = 33.3 min. HR-QToF (ESI) m/z: $[M+2H]^{2+}$ Calcd for $C_{72}H_{111}N_{13}O_{18}$ 723.9163; Found 723.9114

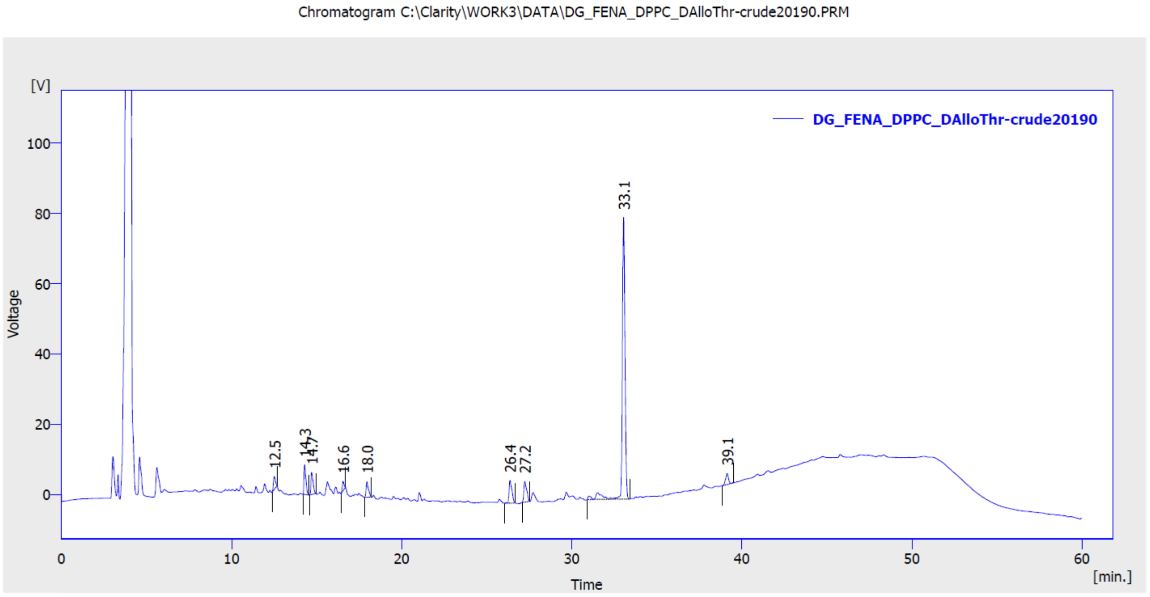

Result Table (Uncal - DG_FENA_DPPC_DAlloThr-crude20190)

	Reten. Time [min]	Area [V.s]	Height [V]	Area [%]	Height [%]	W05 [min]	Compound Name
1	12.520	29.977	3.681	2.3	3.0	0.14	
2	14.303	64.058	8.519	4.8	7.0	0.12	
3	14.713	54.102	6.357	4.1	5.2	0.14	
4	16.560	18.362	2.686	1.4	2.2	0.13	
5	17.963	45.856	4.558	3.5	3.7	0.16	
6	26.377	72.897	6.509	5.5	5.4	0.19	
7	27.247	68.685	5.869	5.2	4.8	0.20	
8	33.053	934.542	80.249	70.5	66.0	0.17	
9	39.140	37.792	3.134	2.8	2.6	0.19	
	Total	1326.273	121.561	100.0	100.0		

Figure S26: Analytical HPLC trace at λ = 220 nm of crude peptide **17C**.

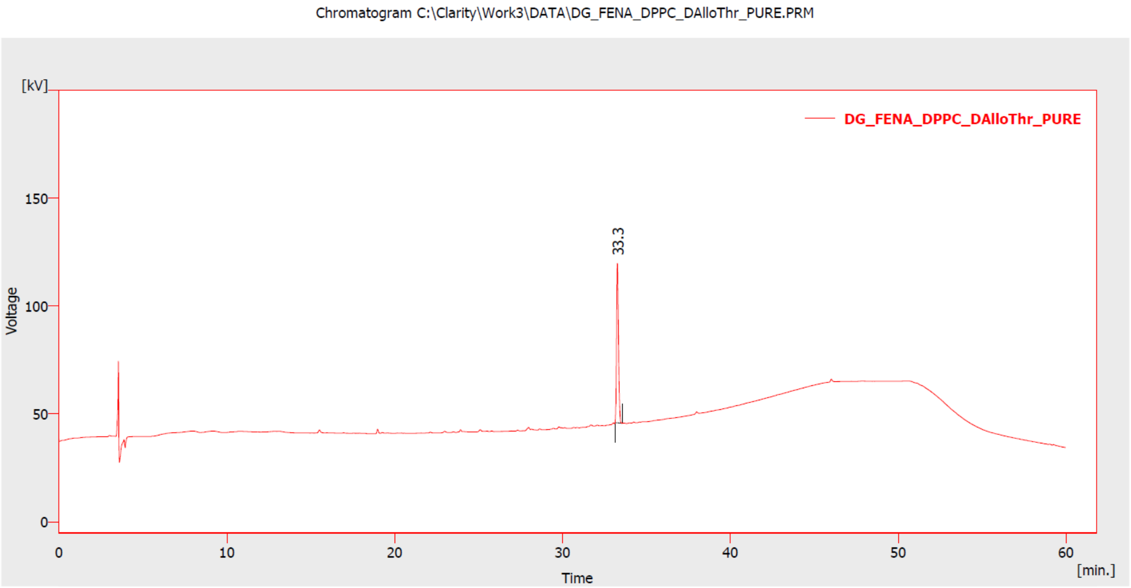

Figure S27: Analytical HPLC trace at λ = 220 nm of purified peptide **17C**.

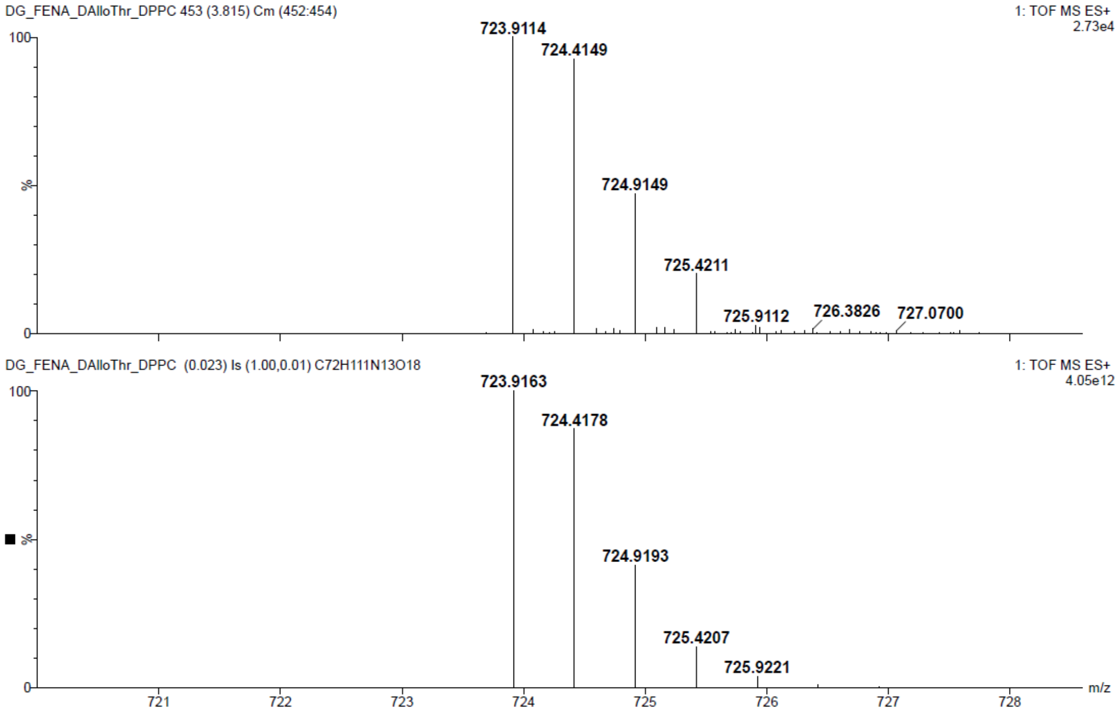

Figure S28: HR-QToF(ESI+)MS analysis of purified peptide **17C**, confirming its identity. Upper panel: calculated mass; lower panel: experimental mass.

Cyclic lipopeptide 17D:

Cyclic lipopeptide **17D** was synthetized in 48% purity. From the crude material, 16.7 mg of cyclic lipodepsipeptide **17D** was isolated in purity >98% (29% yield). HPLC (λ = 220 nm) tR = 32.1 min. HR-QToF (ESI) m/z: [M+2H]2+ Calcd for C72H111N13O18 723.9163; Found 723.9127

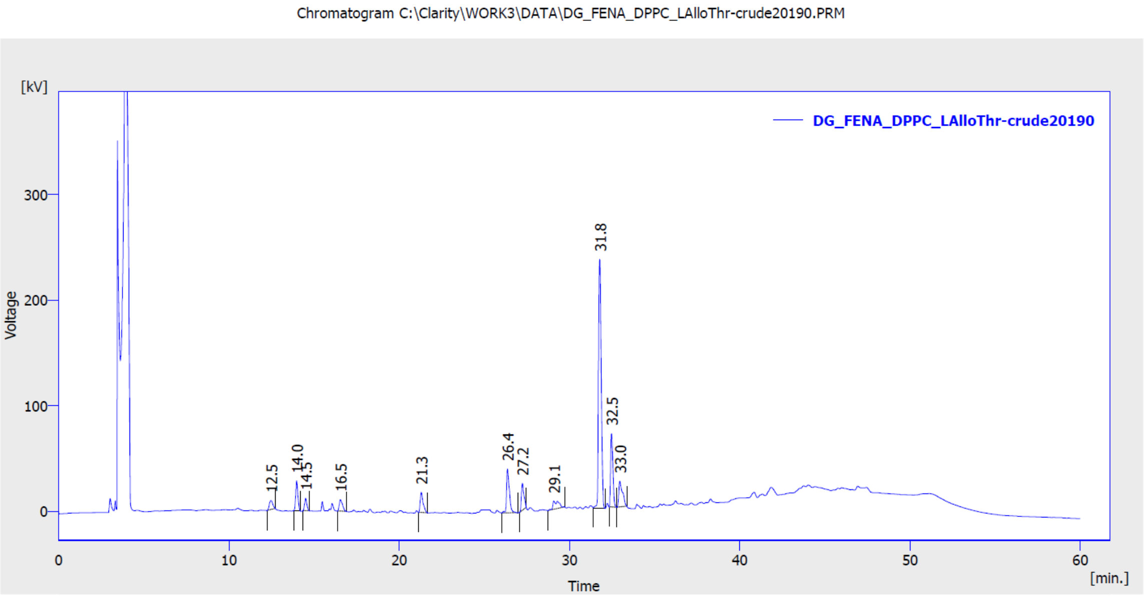

Result Table (Uncal - DG_FENA_DPPC_LAlloThr-crude20190)

	Reten. Time [min]	Area [kV.s]	Height [kV]	Area [%]	Height [%]	W05 [min]	Compound Name
1	12.477	119.551	8.595	2.2	1.8	0.23	
2	13.983	249.882	28.215	4.7	5.8	0.14	
3	14.507	102.751	11.935	1.9	2.5	0.13	
4	16.547	145.363	11.001	2.7	2.3	0.22	
5	21.297	227.384	19.158	4.3	4.0	0.17	
6	26.360	473.934	41.448	8.9	8.6	0.17	
7	27.233	270.594	25.586	5.1	5.3	0.18	
8	29.080	181.117	7.971	3.4	1.6	0.46	
9	31.780	2550.406	235.834	47.9	48.7	0.17	
10	32.473	633.112	69.670	11.9	14.4	0.15	
11	32.957	367.691	24.466	6.9	5.1	0.29	
	Total	5321.784	483.878	100.0	100.0		

Figure S29: Analytical HPLC trace at λ = 220 nm of crude peptide **17D**.

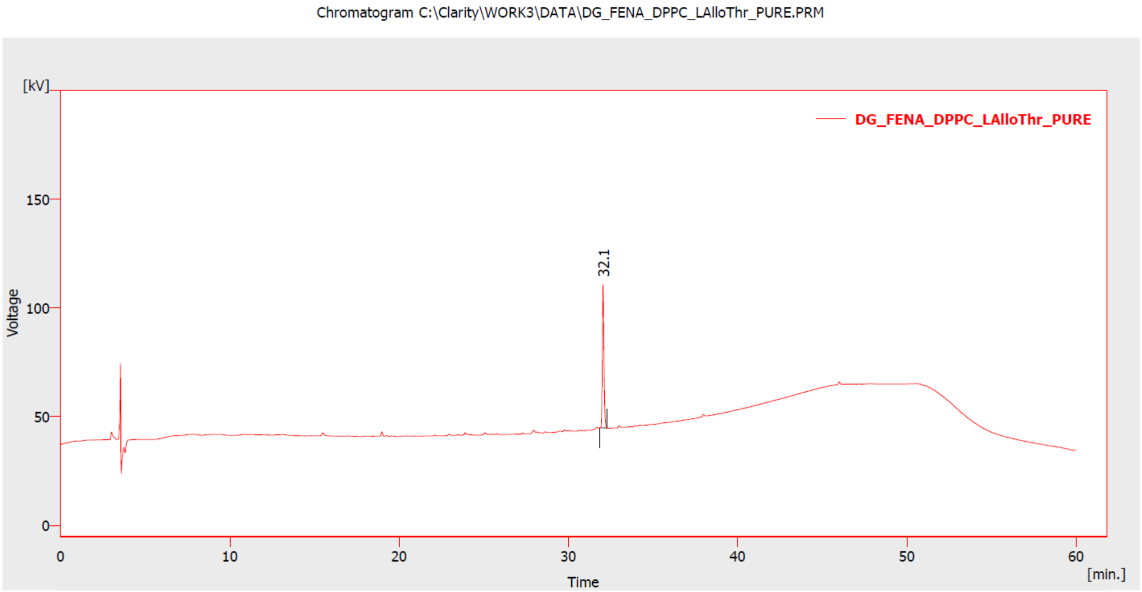

Figure S30: Analytical HPLC trace at λ = 220 nm of purified peptide **17D**.

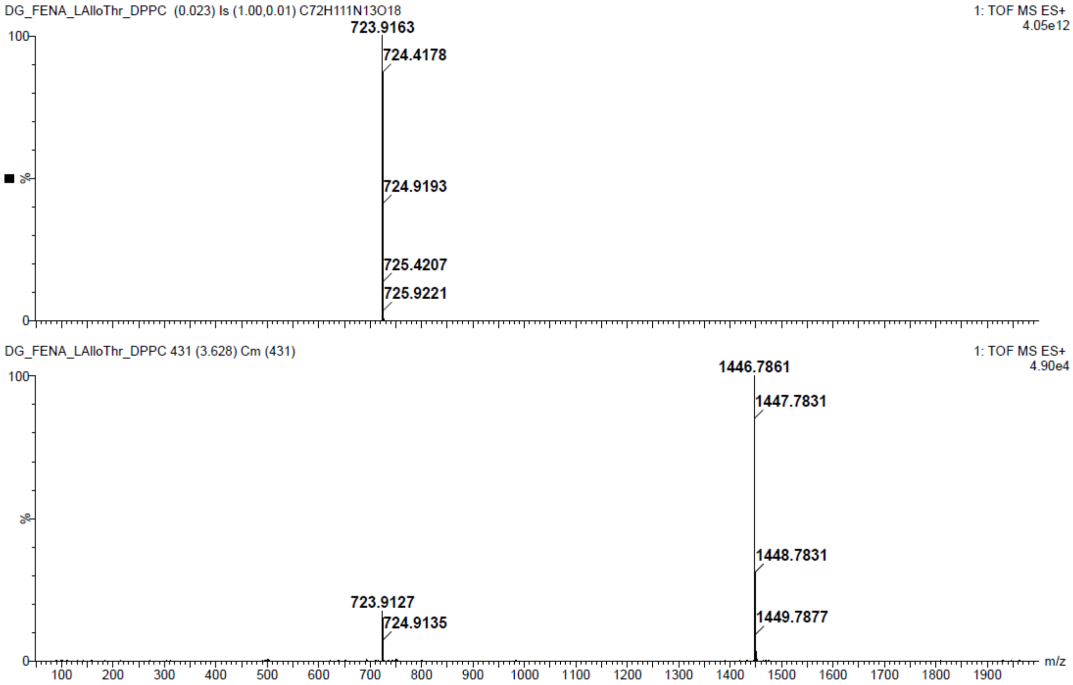

Figure S31: HR-QToF(ESI+)MS analysis of purified peptide **17D**, confirming its identity. Upper panel: calculated mass; lower panel: experimental mass.

Chemical Stability Studies

Natural Fengycin isolation and characterization

In order to compare the chemical stability of compounds **17A-17D** with that of the natural product, 5 mg of Fengycin complex from *Bacillus subtilis* strain F-29-3 were purchased from Aldrich (90% purity) and the mixture purified by RP-HPLC. Fractions corresponding to single chromatographic peaks were then combined together, freeze-dried and analysed by HPLC and LC/(ESI+)MS. As shown below, a broad range of Fengycin isomorfs were found upon analysis (**Figure S32**). Tandem MS/MS spectrometry was then employed to identify the structure of the major product, which corresponded to C_{16}-Fengycin B (purity > 98%). C_{16}-Fengycin A could be also identified and isolated from the mixture; however only marginal amounts could be recovered. For its comparison, characterization data gathered for both lipopeptides is given next.

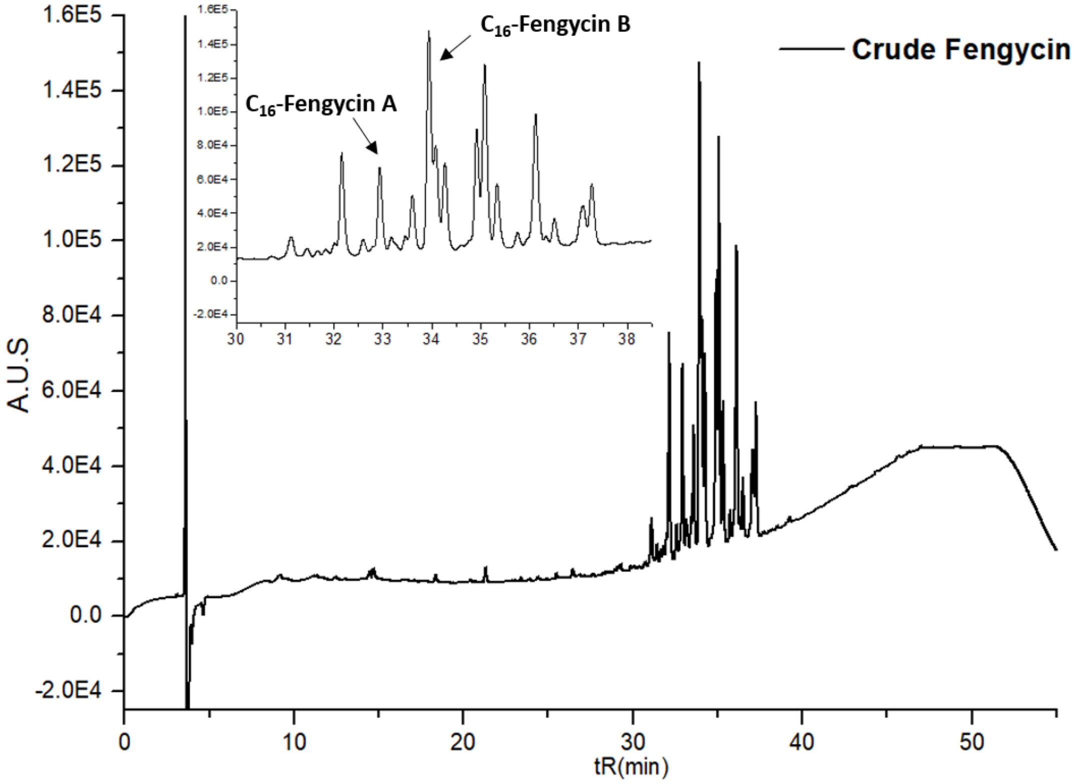

Figure S32: Analytical HPLC analysis of crude Fengycin from Bacillus *ssp* showing the presence of C_{16}-Fengycin B as the main isomorph in the mixture, and only a minor content in C_{16}-Fengycin A.

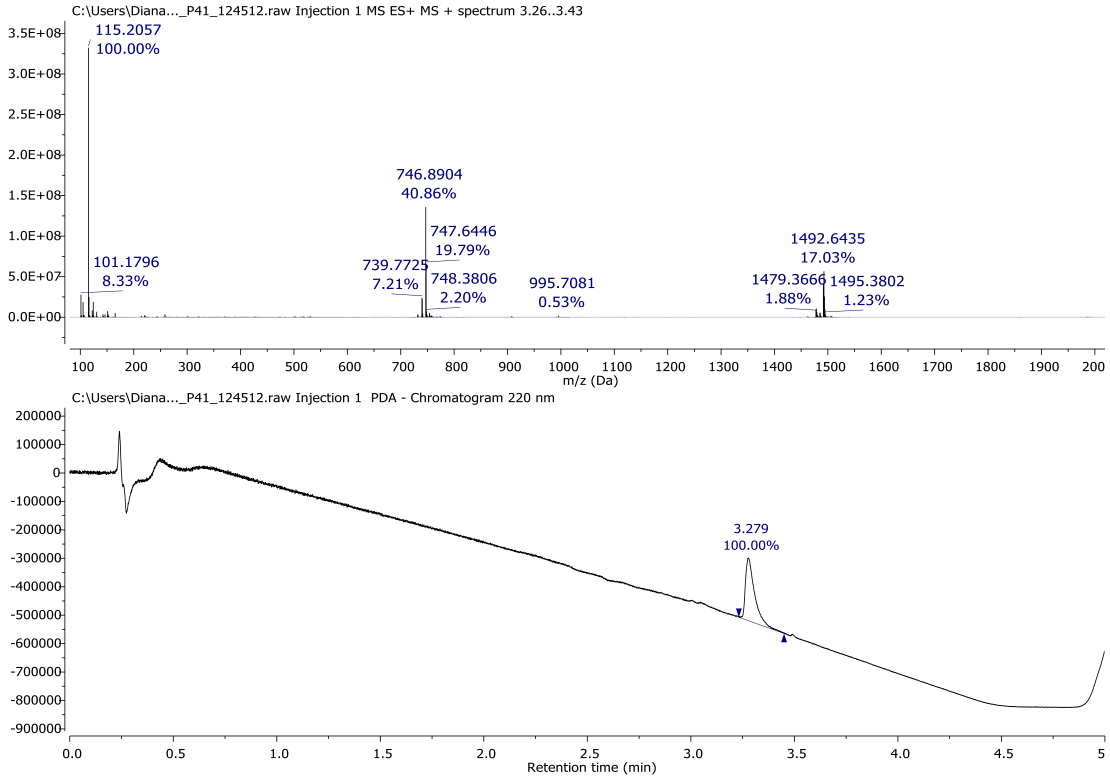

Figure S33: LC/(ESI+)MS analysis of isolated (C_{16})-Fengycin B.

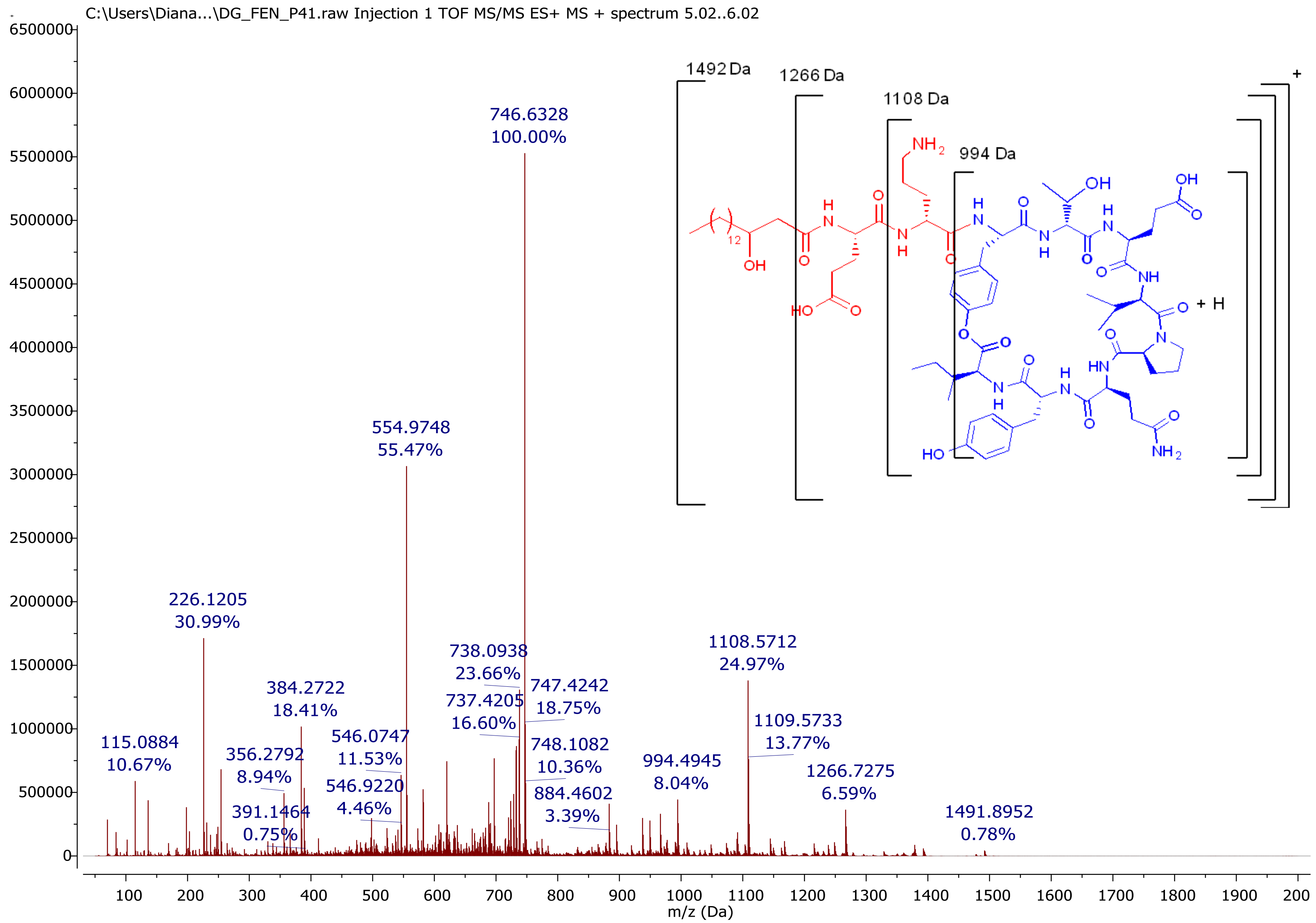

Figure S34: HR-QToF(ESI+) Tandem MS/MS analysis of the purified natural peptide, which confirms its identity as (C_{16})-Fengycin B. Inset panel shows the assignation of the main fragments observed in the higher mass region.

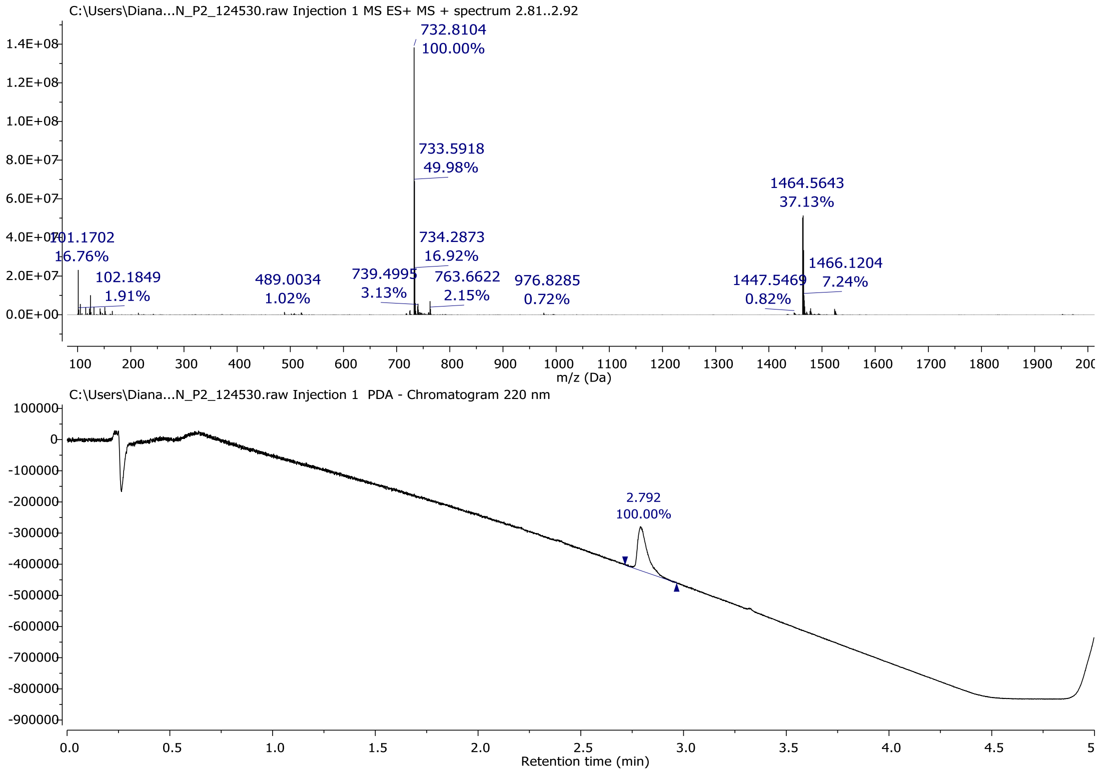

Figure S35: LC/(ESI+)MS analysis of isolated (C_{16})-Fengycin A.

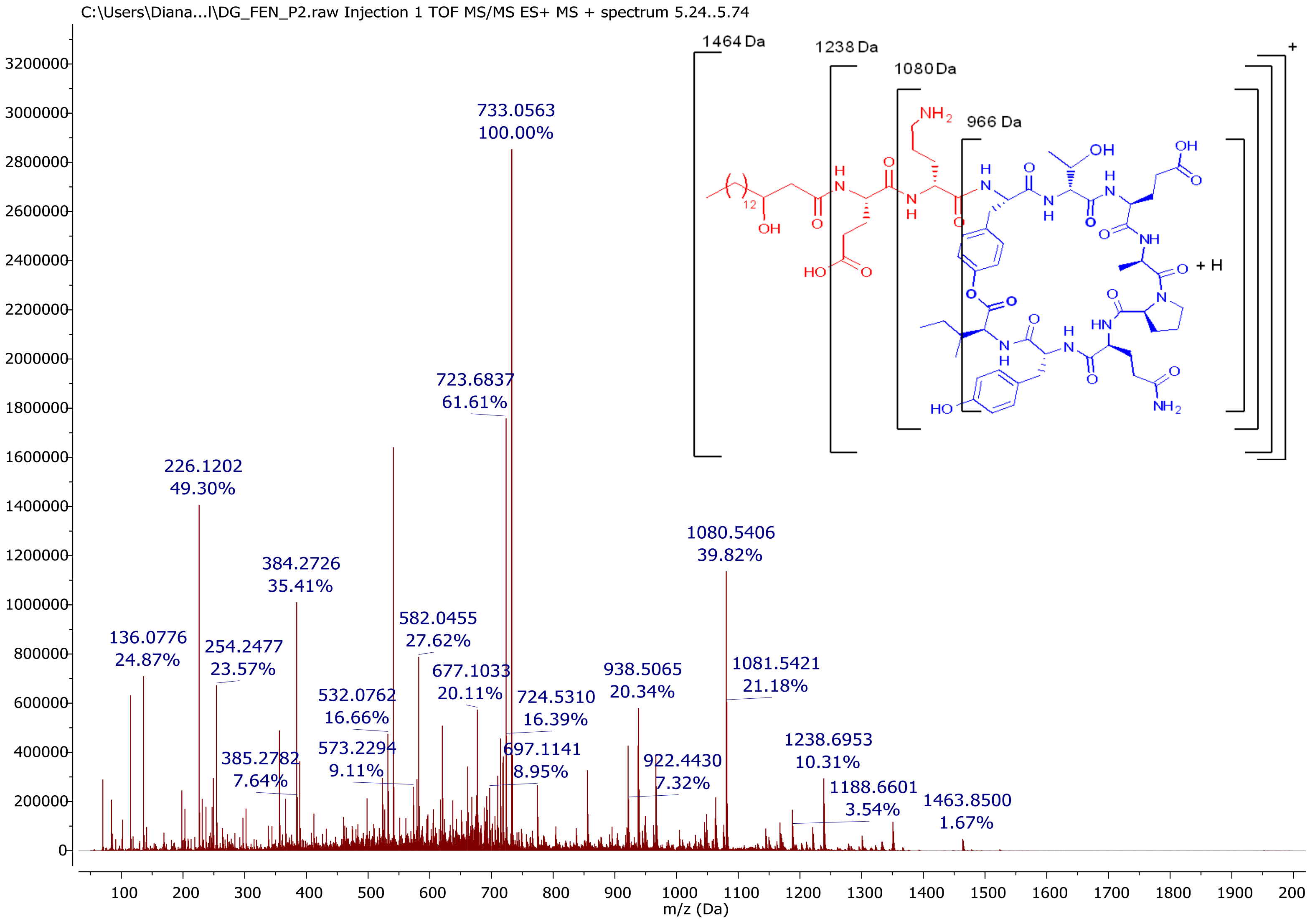

Figure S36: HR-QToF(ESI+) tandem MS/MS analysis of the purified minor product, which confirms its identity as (C_{16})-Fengycin A. Inset panel shows the assignation of the main fragments observed in the higher mass region.

General procedure for sample hydrolysis and sample analysis

To assess the chemical stability of peptides **17A -17D** and also of isolated natural C_{16}-Fengycin B, 30 µL of a stock solution of each peptide in H_2O (1 mg ml^{-1}) was added to an Eppendorf tube containing 120 µL of deionized H_2O. Then 20 µL of a known control peptide (1mg ml^{-1}) was spiked into the sample to be used as an internal standard (tR = 21.9 min) and 50 µL of the mixture kept aside for reference purposes (control samples at t = 0). Note that such control was selected in light of the well reported higher chemical stabilities exhibited by these molecules.[3] The bulk remaining sample was then mixed with 120 µL of either NaOH 100 mM in H_2O (basic hydrolysis) or TFA (acidolytic hydrolysis). Samples this way prepared were then let to incubate at room temperature in a rotary shaker. At each given time, a sample aliquot of 50 µL was taken out from the solution and placed directly in a HPLC vial fitted with a 200 µL conical insert. Then 50 µL of H_2O/MeCN (1:1) were added and the sample immediately analyzed by analytical HPLC. For each peptide, two independent replicas were performed. While typically complete degradation of natural Fengycin in basic conditions could be achieved within a few minutes (Figure S36-S37), extended incubation periods were needed to induce significant levels of degradation in the case of lipopeptides **17A-17D** (S38-S45). Under acidic conditions all samples have been analyzed after a similar incubation period of 12 H. Reported % of undegraded peptide have in all cases been calculated by using the corresponding relative integrated chromatographic areas, at λ = 220 nm, before and after incubation.

HPLC data

Natural C_{16}-Fengycin B:

Natural (C16)-Fengycin B was corroborated to be extremely sensitive to basic conditions. Indeed, direct HPLC analysis of a sample left for incubation for a period as short as 5 minutes revealed complete degradation of the product, as proved by the total disappearance of its corresponding HPLC peak (tR = 33.4 min) and the formation of a complex mixture of multiple by-products. (Figure S37).

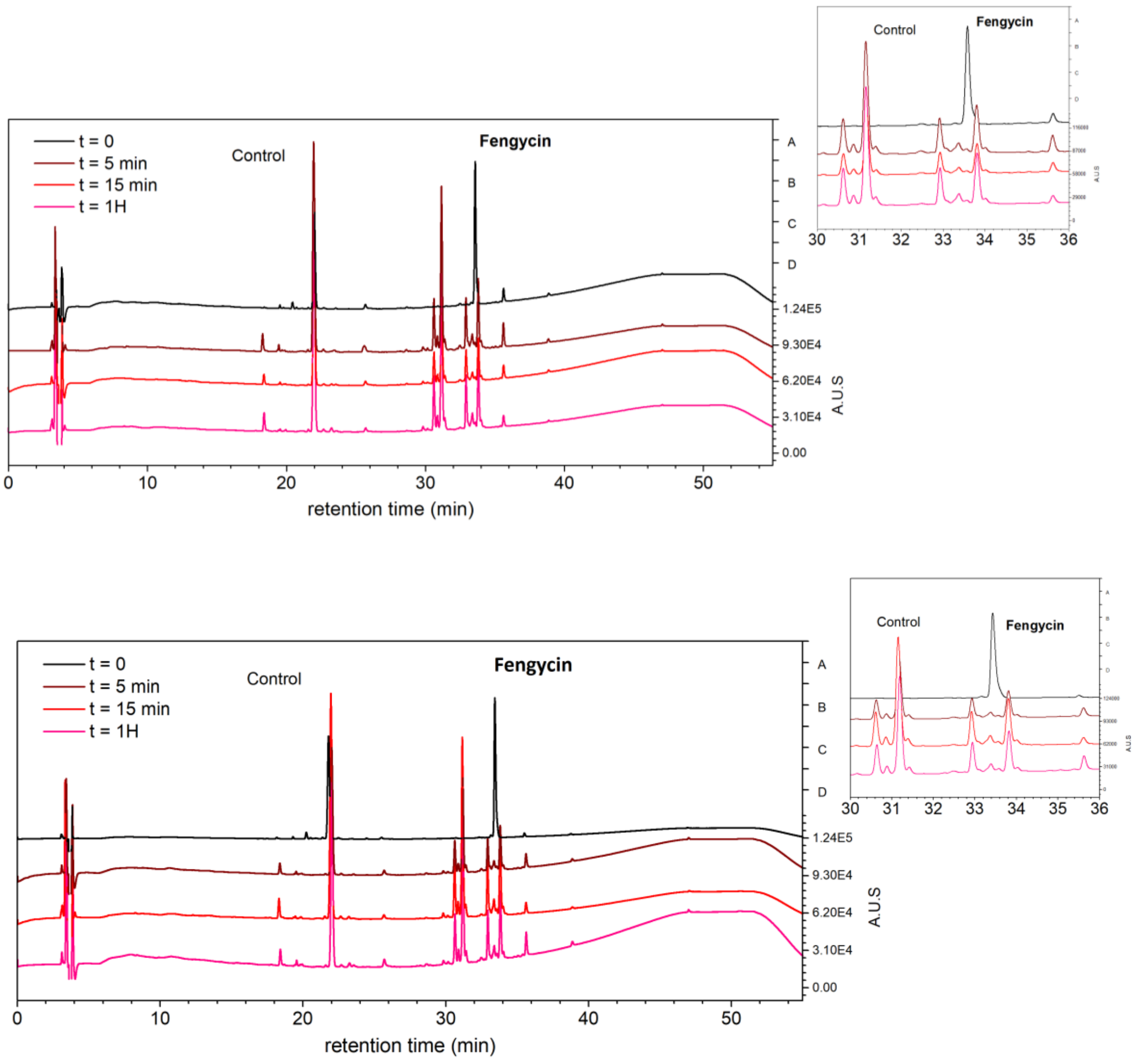

Figure S37: Duplicate analytical RP-HPLC analysis (λ = 220 nm) showing the time-dependent hydrolysis of natural C_{16}-Fengycin B under base catalysed conditions.

Acidic hydrolysis (TFA/H_2O):

As could be expected, the natural product showed better stability under acidic conditions, requiring an incubation time of 12 h at room temperature to achieve full peptide degradation (Figure S38).

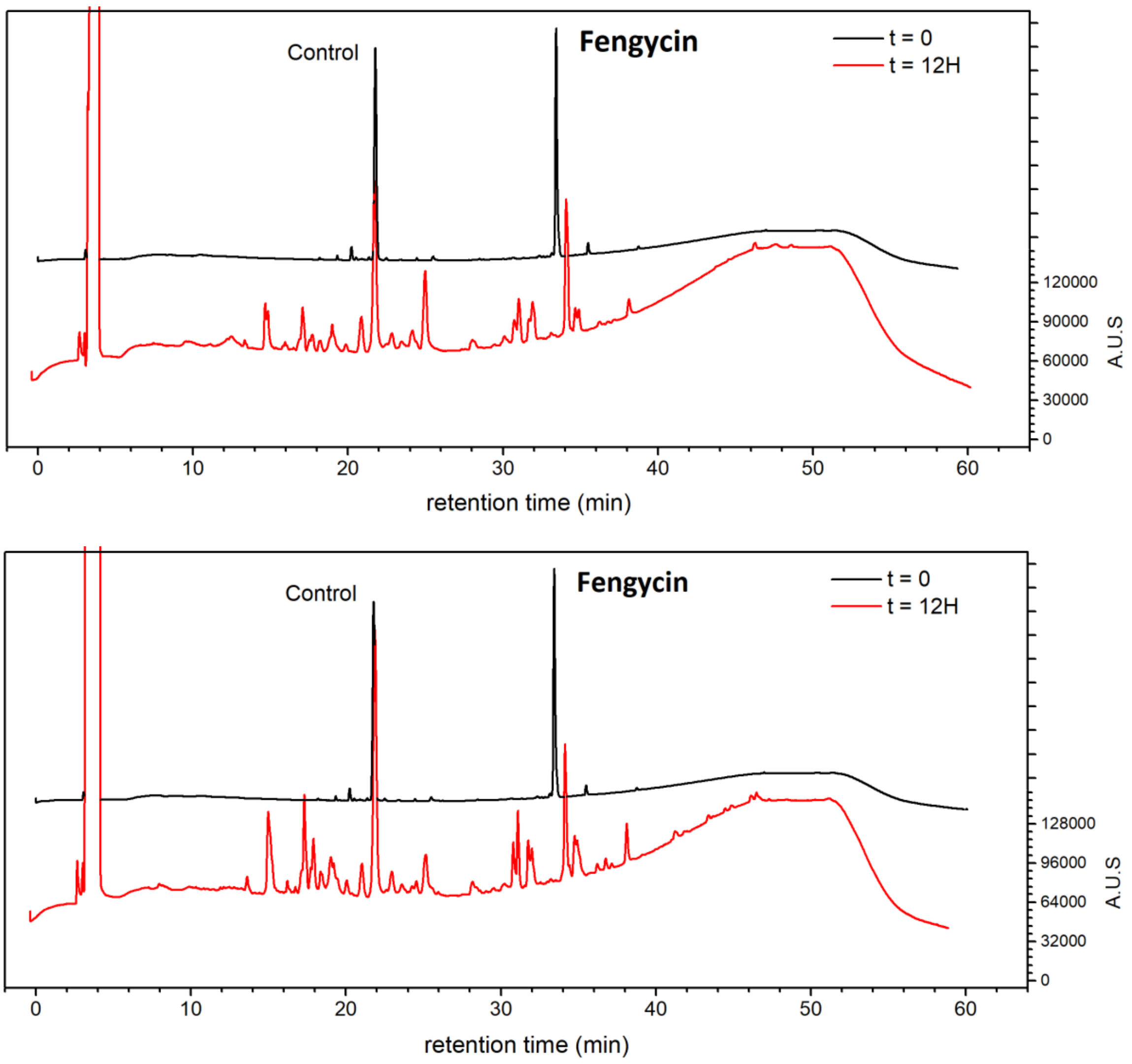

Figure S38: Duplicate analytical RP-HPLC analysis (λ = 220 nm) showing the time-dependent hydrolysis of natural C_{16}-Fengycin B under acid catalysed conditions.

Cyclic lipopeptide 17A (DThr):

Basic hydrolysis (NaOH 50 mM):

HPLC analysis of the samples before and after 18 H of incubation in NaOH 50 mM showed [81-82]% of undegraded peptide **17A** still present, as based in the peaks analytical areas at λ= 220 nm.

A) 82% undegraded peptide

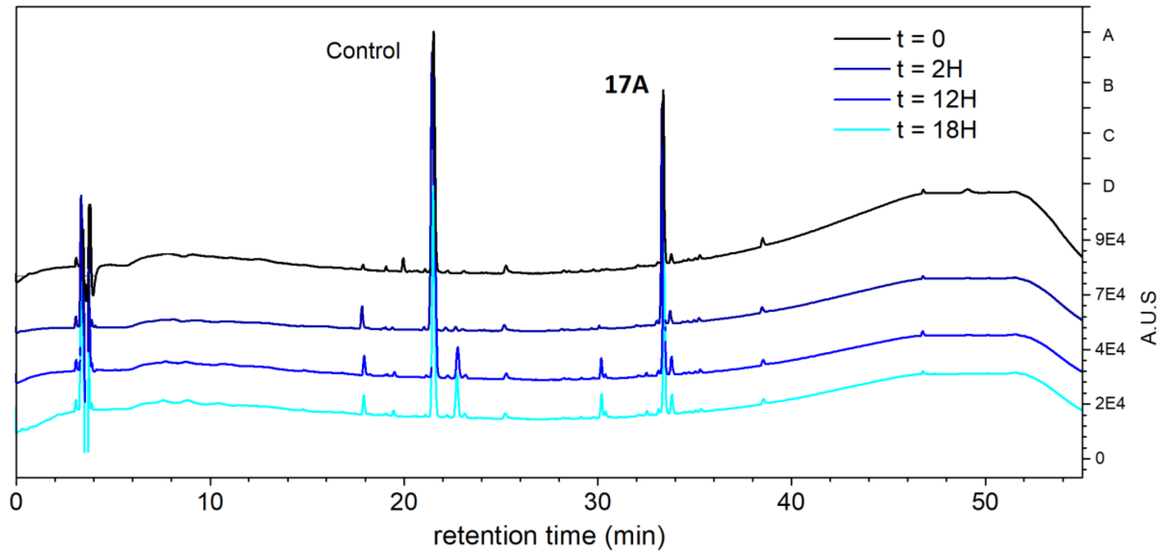

B) 81% undegraded peptide

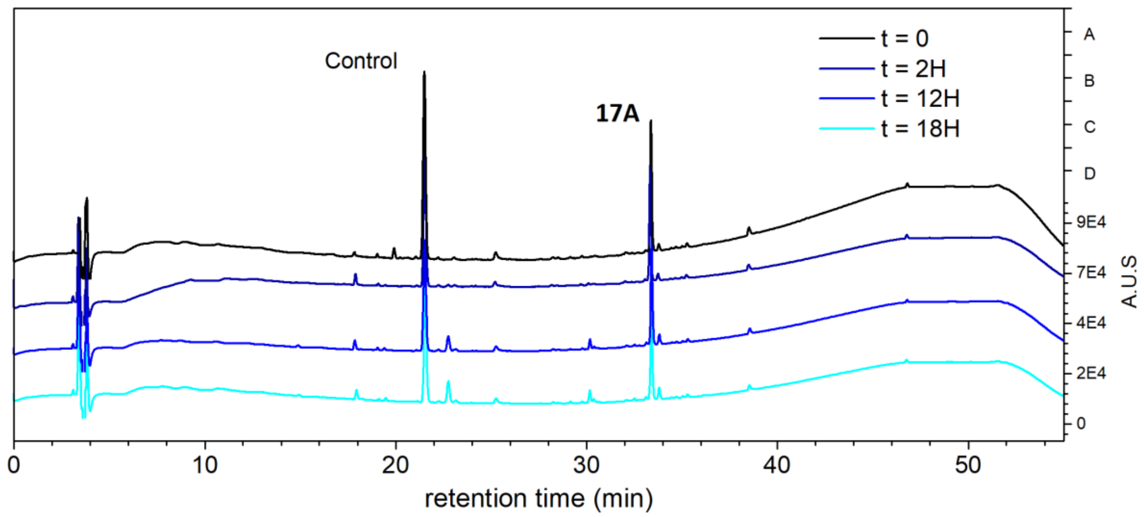

Figure S39: Duplicate analytical RP-HPLC analysis (λ = 220 nm) showing the time-dependent hydrolysis of modified Fengycin analogue **17A** under base catalysed conditions.

Acidic hydrolysis (TFA/H_2O):

HPLC analysis of the samples before and after 12 H of incubation in TFA/H_2O 1:1 v/v mM showed [77-83]% of undegraded peptide **17A**.

A) 83% undegraded peptide

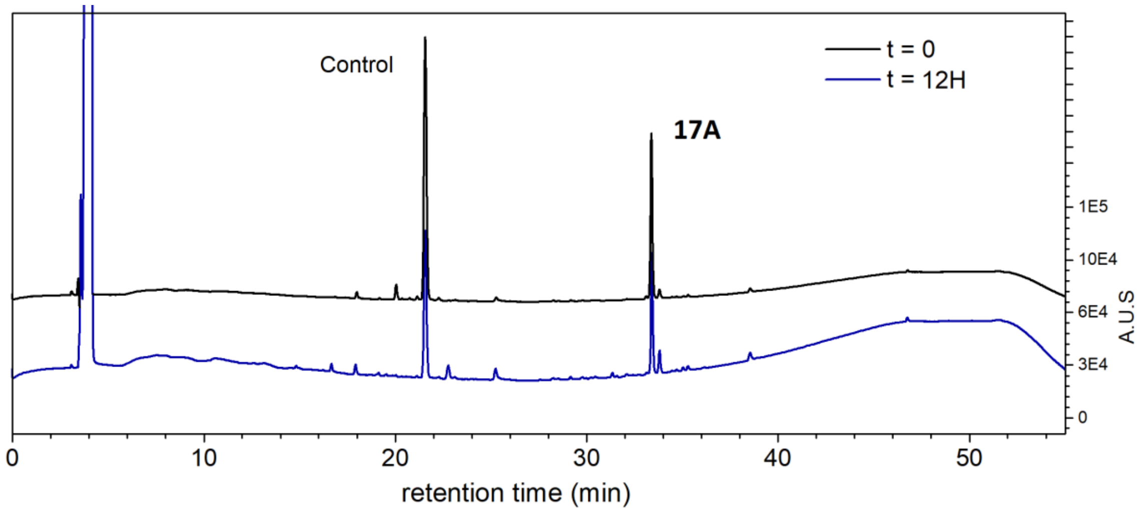

B) 77% undegraded peptide

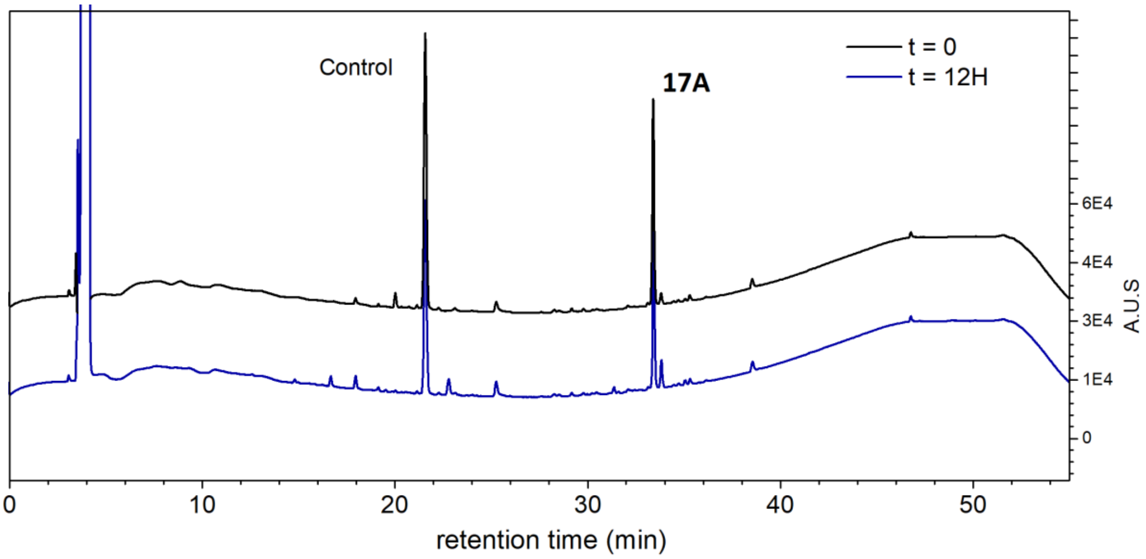

Figure S40: Duplicate analytical RP-HPLC analysis (λ = 220 nm) showing the time-dependent hydrolysis of modified Fengycin analogue **17A** under acid catalysed conditions.

Cyclic lipopeptide 17B LThr:

Basic hydrolysis (NaOH 50 mM):

HPLC analysis of the samples before and after 18 H of incubation in NaOH 50 mM showed [78-82]% of the undegraded peptide **17B** still present.

A) 82% undegraded peptide

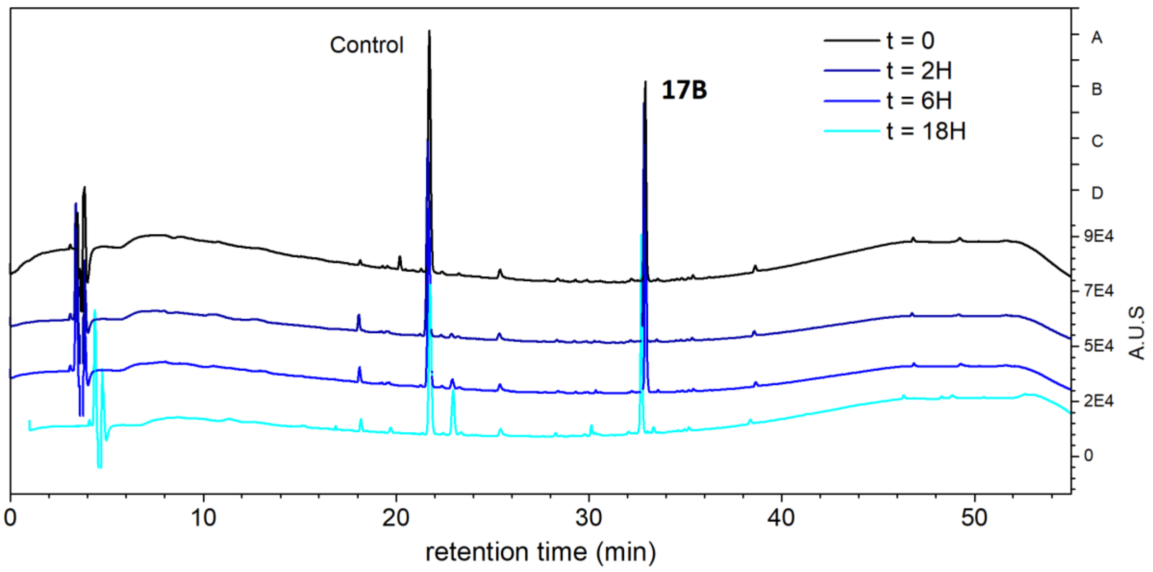

B) 78% undegraded peptide

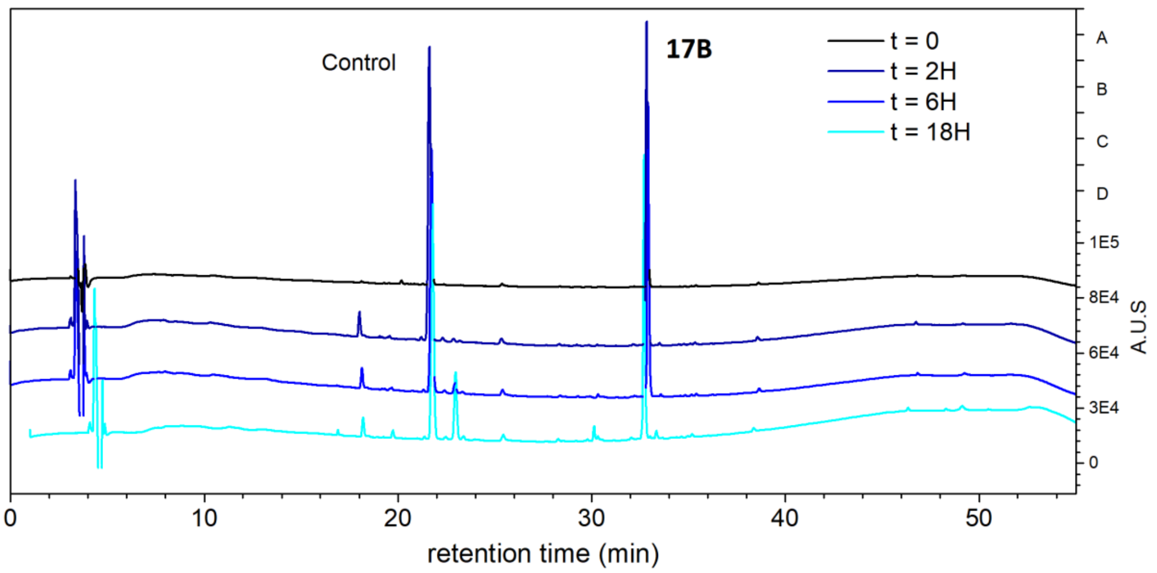

Figure S41: Duplicate analytical RP-HPLC analysis (λ = 220 nm) showing the time-dependent hydrolysis of modified Fengycin analogue **17B** under base catalysed conditions.

Acidic hydrolysis (TFA/H_2O):

HPLC analysis of the samples before and after 12 H of incubation in TFA/H_2O 1:1 v/v mM showed [76-78]% of undegraded peptide **17B**.

A) 78% undegraded peptide

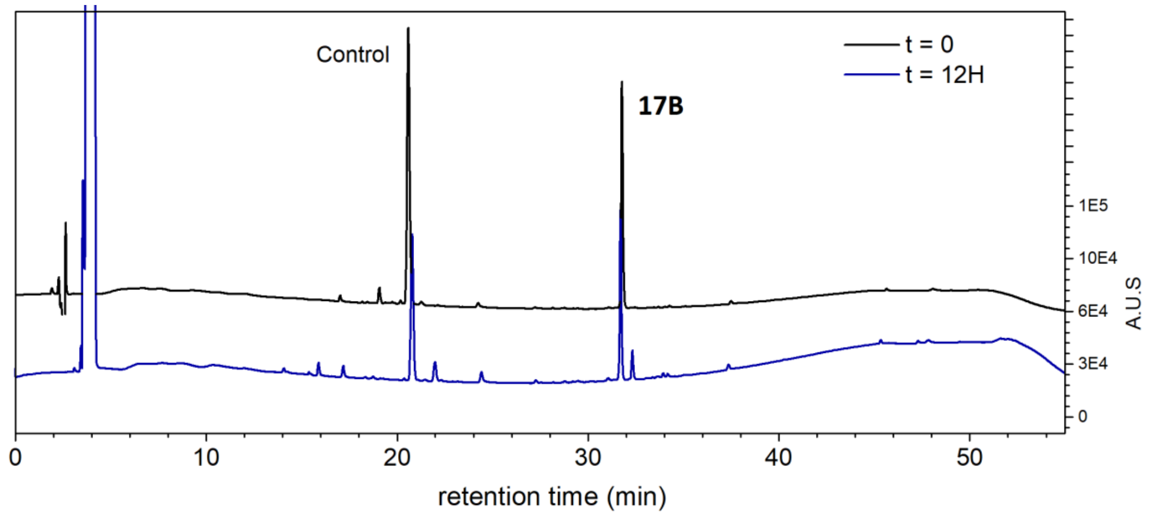

B) 76% undegraded peptide

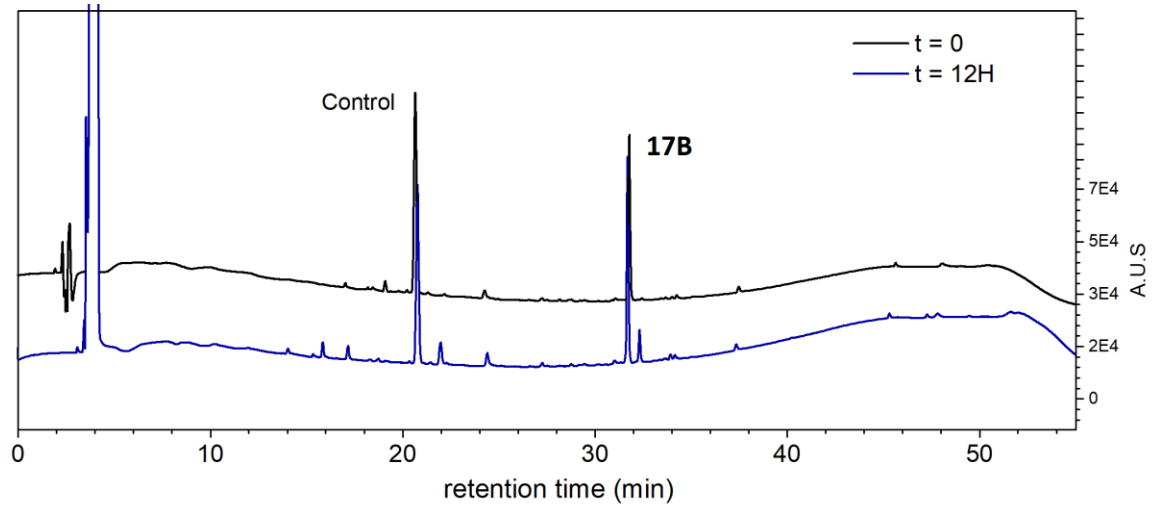

Figure S42: Duplicate analytical RP-HPLC analysis (λ = 220 nm) showing the time-dependent hydrolysis of modified Fengycin analogue **17B** under acid catalysed conditions.

Cyclic lipopeptide 17C DAlloThr:

Basic hydrolysis (NaOH 50 mM):

HPLC analysis of the samples before and after 24 H of incubation in NaOH 50 mM showed [77-75]% of the undegraded peptide **17B** still present.

A) 77% undegraded peptide

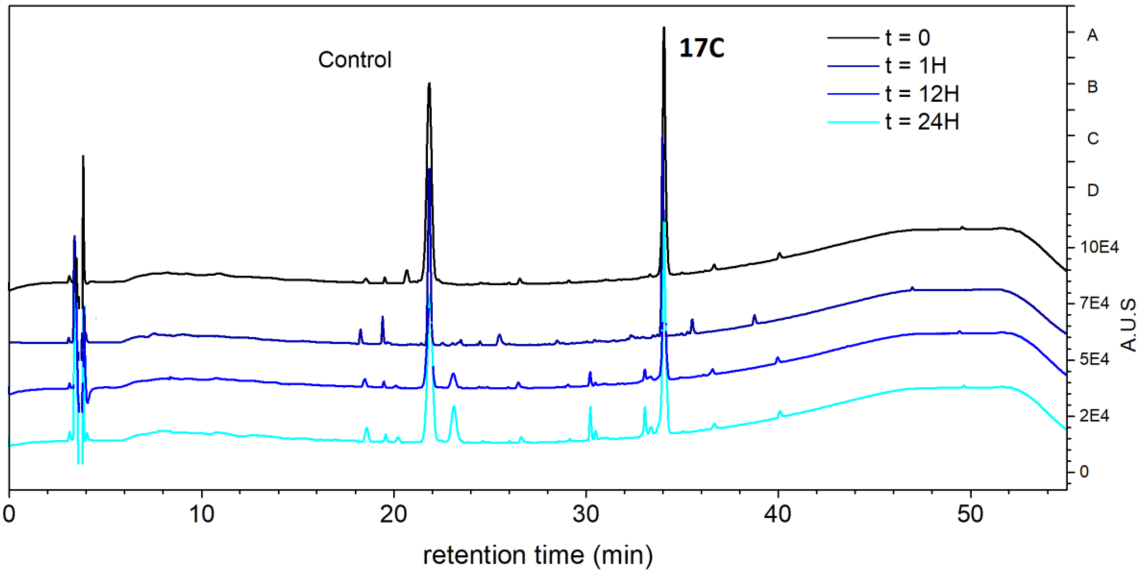

A) 75% undegraded peptide

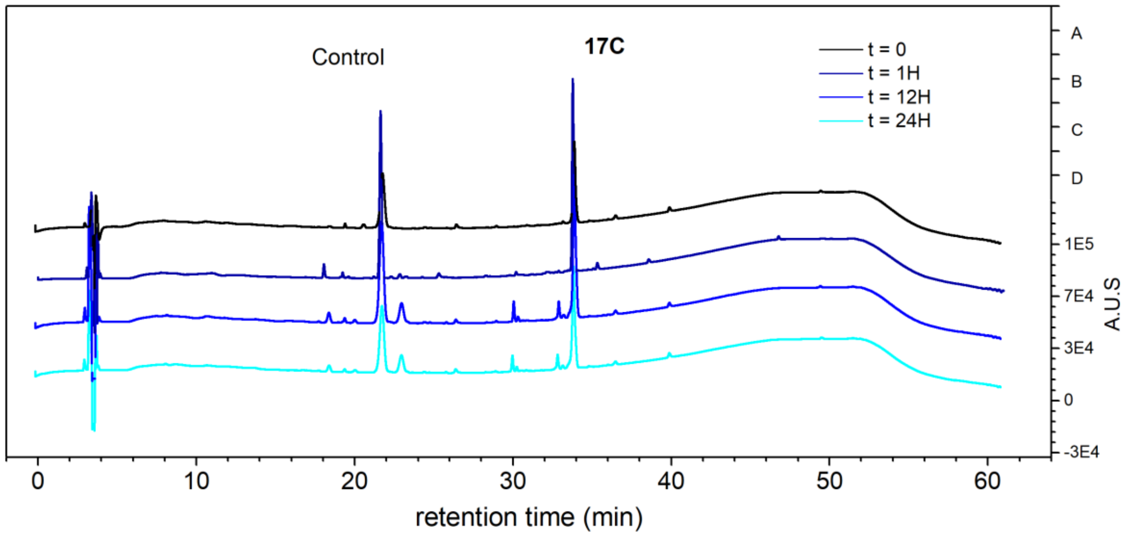

Figure S43: Duplicate analytical RP-HPLC analysis (λ = 220 nm) showing the time-dependent hydrolysis of modified Fengycin analogue **17C** under base catalysed conditions.

Acidic hydrolysis (TFA/H_2O):

HPLC analysis of the samples before and after 12 H of incubation in TFA/H_2O 1:1 v/v mM showed 91% of undegraded peptide **17C**.

A) 91% undegraded peptide

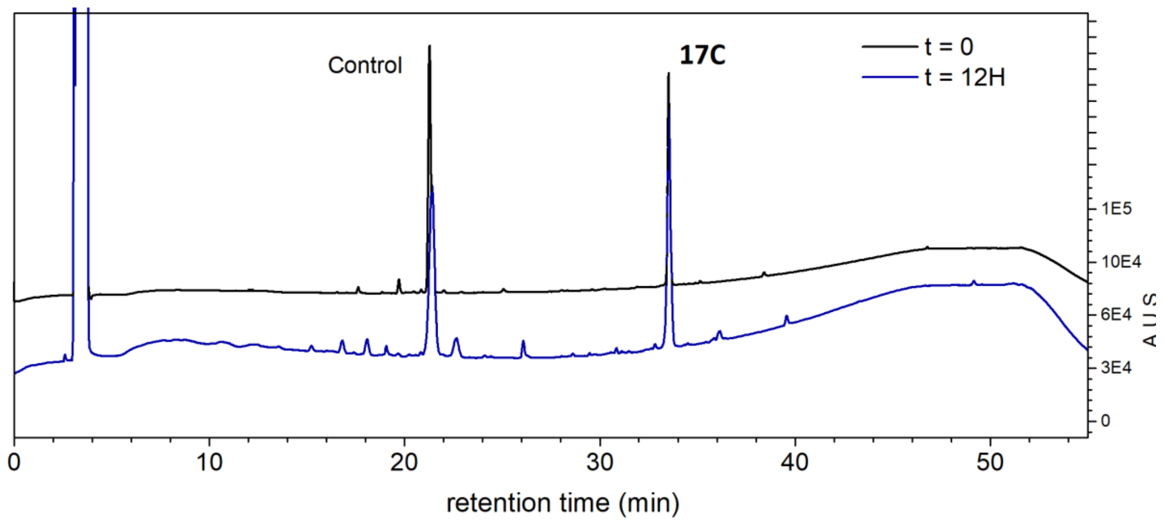

B) 91% undegraded peptide

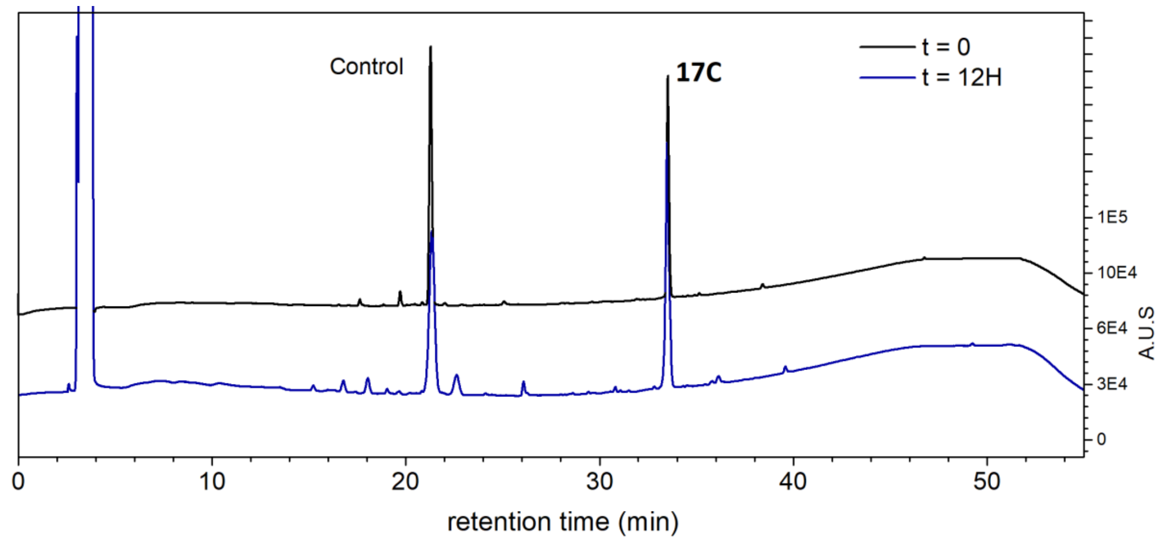

Figure S44: Duplicate analytical RP-HPLC analysis (λ = 220 nm) showing the time-dependent hydrolysis of modified Fengycin analogue **17C** under acid catalysed conditions.

Cyclic lipopeptide 17D LAlloThr:

Basic hydrolysis (NaOH 50 mM):

HPLC analysis of the samples before and after 18 H of incubation in NaOH 50 mM showed [94-96]% of undegraded peptide **17D** still present.

A) 96% undegraded peptide

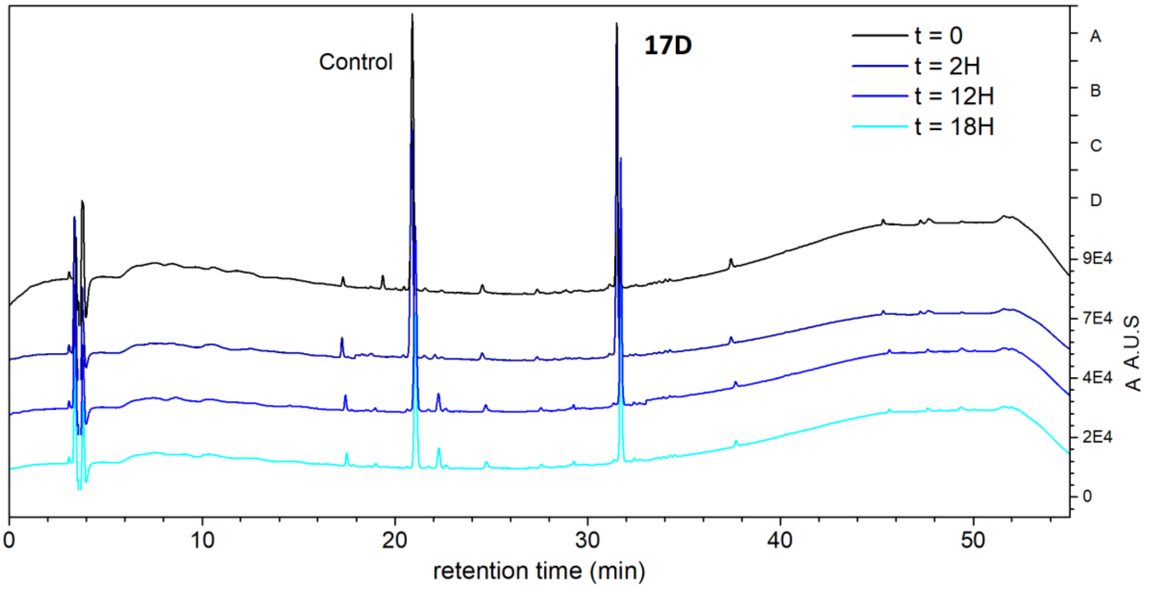

B) 94% undegraded peptide

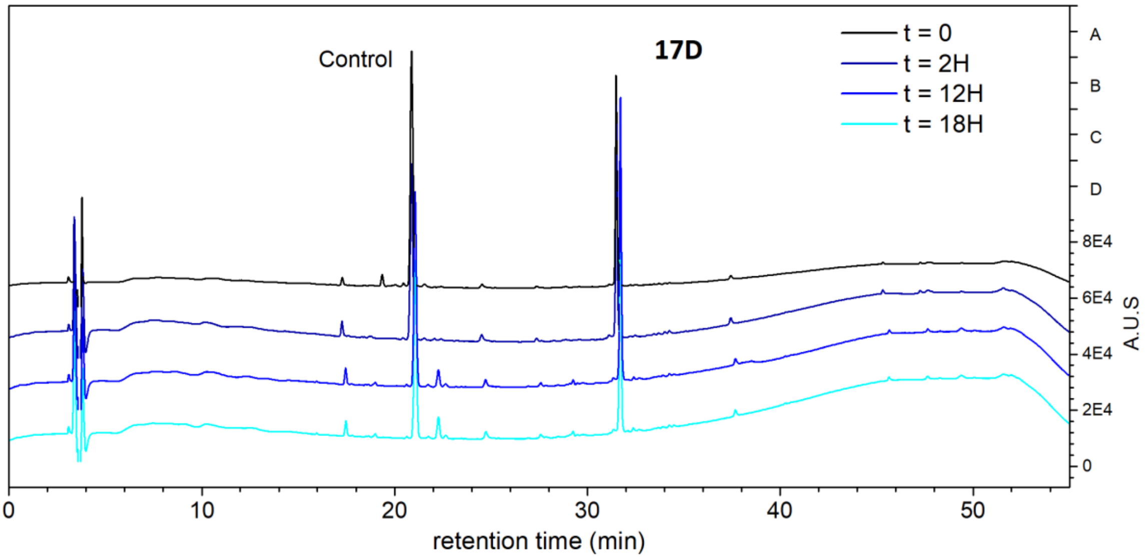

Figure S45: Duplicate analytical RP-HPLC analysis (λ = 220 nm) showing the time-dependent hydrolysis of modified Fengycin analogue **17D** under base catalysed conditions.

Acidic hydrolysis (TFA/H_2O):

HPLC analysis of the samples before and after 12 H of incubation in TFA/H_2O 1:1 v/v mM showed [83-84]% of undegraded peptide **17D**.

A) 84% undegraded peptide

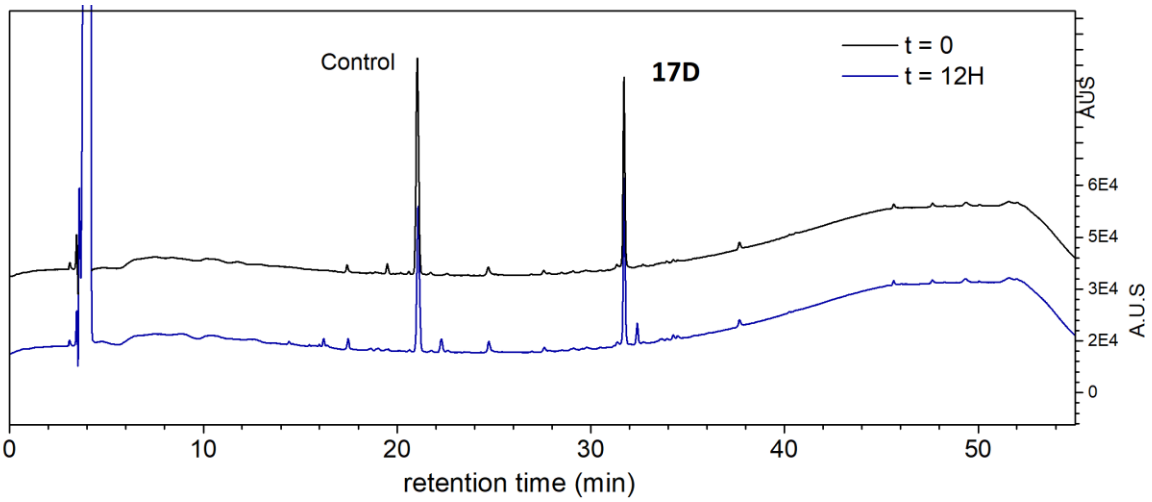

B) 83% undegraded peptide

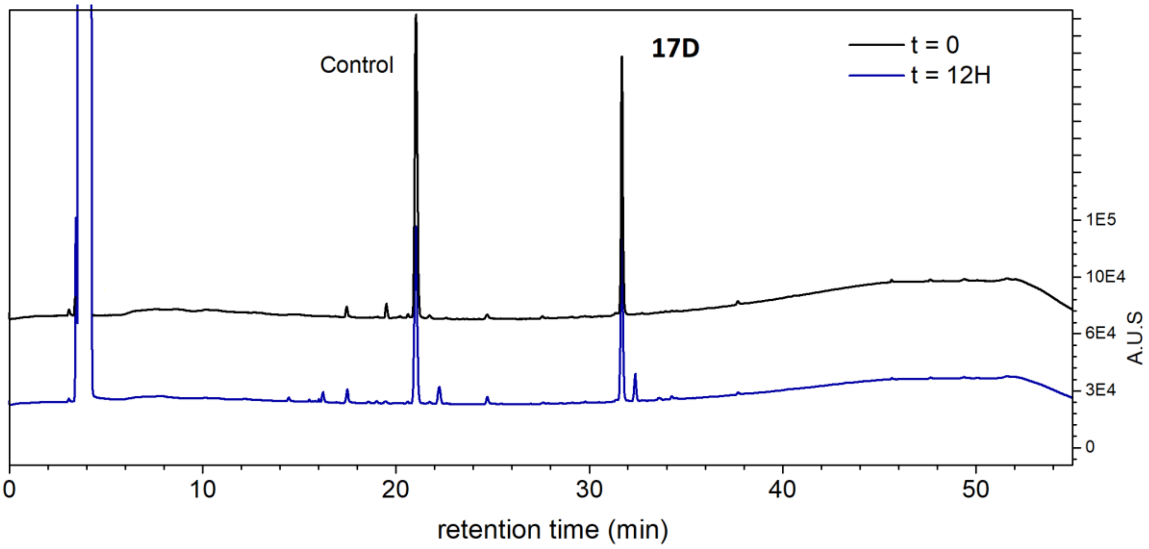

Figure S46: Duplicate analytical RP-HPLC analysis (λ = 220 nm) showing the time-dependent hydrolysis of modified Fengycin analogue **17D** under acid catalysed conditions.

Microbiological experiments

Chemicals and microorganisms

Fusarium graminearum (ATCC 36016) was obtained from DSMZ- German Collection of Microorganisms and Cell Culture GmBH and maintained on malt extract agar. *Bacillus* sp. CS93 (NRRL β-21974) was acquired from the Microbial Genomics and Bioprocessing Research Unit, National Centre for Agricultural Utilization Research, Peoria, IL, USA and maintained on tryptic soy agar. The bacterium was grown in media optimal for lipopeptide production (MOLP) for 72 hours as described in O'Connor *et al*.[4] and the natural fengycin isolated from the culture supernatant by acid precipitation, solid phase extraction (Hypersep C-18, Supelco) and HPLC (Varian Pro-Star). Purified fengycins were finally eluted from a Zorbax SB-C18 9.4 x 250 mm column (Agilent) using a gradient elution from 10-100 % acetonitrile containing 0.1 % TFA. Elution was over 75 min with a flow rate of 4 mL/min; fractions containing fengycin were identified by mass spectrometry and shown to contain fengycins with chain lengths ranging from C15-C20. These were pooled, dried and dissolved in water for bioassay.

Bioassays

F. graminearum was inoculated in sabouraud dextrose broth containing either natural or synthetic fengycin (500 µg/mL) in 96 well plates. A control experiment in which the fungus was incubated in the absence of fengycin was also conducted. Fungal growth was assessed by measuring the absorbance (600 nm) every 24 hours using an Epoch Microplate Spectrophotometer and Take3 plate (Biotek). All experiments were carried out in duplicate.

Additional References

(1) Kaiser, E.; Colescott, R. L.; Bossinger, C. D.; Cook, P. I. Color Test for Detection of Free Terminal Amino Groups in the Solid-Phase Synthesis of Peptides. *Anal. Biochem.* **1970**, *34* (2), 595–598. https://doi.org/10.1016/0003-2697(70)90146-6.

(2) Conroy, T.; Jolliffe, K. A.; Payne, R. J. Efficient Use of the Dmab Protecting Group: Applications for the Solid-Phase Synthesis of N-Linked Glycopeptides. *Org. Biomol. Chem.* **2009**, *7* (11), 2255–2258. https://doi.org/10.1039/B821051A.

(3) Rosales, A. M.; Murnen, H. K.; Zuckermann, R. N.; Segalman, R. A. Control of Crystallization and Melting Behavior in Sequence Specific Polypeptoids. *Macromolecules* **2010**, *43* (13), 5627–5636. https://doi.org/10.1021/ma1002563.

(4) O'Connor, N. K.; Hudson, A. S.; Cobb, S. L.; O'Neil, D.; Robertson, J.; Duncan, V.; Murphy, C. D. Novel Fluorinated Lipopeptides from Bacillus Sp. CS93 via Precursor-Directed Biosynthesis. *Amino Acids* **2014**, *46* (12), 2745–2752. https://doi.org/10.1007/s00726-014-1830-z.
